# Supplementary material for: Contribution of epidermal growth factor (EGF) in the treatment of cutaneous leishmaniasis caused by Leishmania major in BALB/c mice
Source: PLoS Negl Trop Dis. 2025 Jan 14;19(1):e0012765. doi: 10.1371/journal.pntd.0012765 (PMC11771879; doi:10.1371/journal.pntd.0012765)
Supplement: S1 Table — (PDF) [file pntd.0012765.s001.pdf]

S1 Table: The wound size of BALB/c mice infected with *L. major* in study groups during five weeks of treatment.

Table A: The average lesion size of study groups in each week after the onset of treatment.

Weeks after the onset of treatment	S+E4.5			S+E1.5			S			G+E4.5			G+E1.5			G			N			C		
	**Average**	**SD**	**n**	**Average**	**SD**	**N**	**Average**	**SD**	**n**	**Average**	**SD**	**n**	**Average**	**SD**	**n**	**Average**	**SD**	**n**	**Average**	**SD**	**n**	**Average**	**SD**	**n**
**0**	7.01	1.14	9	7.28	1.32	9	7.02	1.94	9	7.31	1.03	9	7.85	1.03	9	7.33	1.84	9	8.11	1.23	9	8.10	1.27	9
**1**	7.36	1.07	9	7.73	1.09	9	8.04	1.85	9	8.10	0.75	9	8.2	0.88	9	8.1	1.5	9	8.83	0.95	9	8.81	0.7	9
**2**	8.013	0.91	9	8.61	0.96	9	8.65	1.89	9	8.32	0.85	9	8.89	0.74	9	9.13	1.02	9	10.88	1.08	9	11.29	1.12	9
**3**	8.21	1.01	9	9.46	0.8	9	9.18	1.95	9	9.36	0.87	9	10.52	0.55	9	10.98	1.19	9	12.48	1.02	9	13.56	0.66	8
**4**	7.68	0.63	9	9.95	0.87	9	10.25	1.09	9	9.41	1.21	9	11.83	0.87	9	12.40	0.87	8	15.08	1.03	7	16.29	0.64	6
**5**	6.98	0.78	9	9.81	0.88	9	9.91	0.47	9	8.78	0.82	9	11.94	1.04	9	12.11	0.79	8	18.65	0.77	7	19.86	0.62	5

SD: Standard deviation; **n:** number of mice per group

S+E1.5: SinaAmpholeish gel (daily) with subcutaneous injection EGF 1.5 µg/kg (Alternate day)

S+E4.5: SinaAmpholeish gel (daily) with subcutaneous injection EGF 4.5 µg/kg (Alternate day)

G+E1.5: Intramuscular injection of Glucantime 20 mg/kg (daily) with subcutaneous injection EGF 1.5 µg/kg (Alternate day)

G+E4.5: Intramuscular injection of Glucantime 20 mg/kg (daily) with subcutaneous injection EGF 4.5 µg/kg (Alternate day)

G: Intramuscular injection of Glucantime 20 mg/kg (daily)

S: SinaAmpholeish gel (daily)

N: Subcutaneous injection of Normal Saline (Alternate day)

C: Control without drug treatment

Table B: The wound size in BALB/c mice in each group during five weeks of treatment.

	Lesion size (mm^2)							
**Week 0**	**S+E4.5**	**S+E1.5**	**S**	**G+E4.5**	**G+E1.5**	**G**	**N**	**C**
**Mice**	6.97	7.87	6.98	7.4	6.94	5.08	8.17	9.98
	6.02	6.06	10.67	7.85	8.09	6.81	9.41	7.95
	7.32	7.31	7.18	8.04	6.69	8.49	8.22	8.98
	5.95	5.27	5.06	9.21	8.94	9.13	9.13	9.06
	6.07	8.65	5.14	6.21	6.37	4.09	9.35	8.92
	5.96	7.66	8.95	7.69	7.35	7.82	8.52	7.94
	7.82	5.74	8.12	7.05	8.49	9.86	7.85	7.21
	7.75	9.08	5.53	6.02	8.89	7.04	6.11	5.9
	9.25	7.87	5.58	6.33	8.88	7.61	6.2	6.99
**Mean ± SD**	7.01 ± 1.14	7.28 ± 1.32	7.02 ± 1.94	7.31 ± 1.03	7.85 ± 1.03	7.33 ± 1.84	8.11 ± 1.23	8.10 ±1.27
**Week 1**	**S+E4.5**	**S+E1.5**	**S**	**G+E4.5**	**G+E1.5**	**G**	**N**	**C**
**Mice**	7.52	7.91	7.89	7.91	7.29	6.48	9.1	8.75
	6.45	6.73	11.71	8.18	8.36	8.26	9.65	8.69
	7.41	7.84	8.02	8.35	7.45	8.67	8.92	9.62
	6.37	6.1	6.73	9.79	9.01	9.57	8.53	9.97
	6.61	8.81	6.58	7.05	6.88	5.12	9.94	9.39
	6.28	8.42	9.93	8.11	7.74	8.53	9.8	8.48
	8.11	6.35	8.79	8.2	8.79	9.92	8.77	8.1
	7.96	9.11	6.73	7.75	9.17	7.74	7.19	7.91
	9.54	8.32	6.02	7.55	9.11	8.63	7.61	8.42
**Mean ± SD**	7.36 ± 1.07	7.73 ± 1.09	8.04 ± 1.85	8.10 ± 0.75	8.20 ± 0.88	8.10 ± 1.50	8.83 ± 0.95	8.81 ± 0.70
**Week 2**	**S+E4.5**	**S+E1.5**	**S**	**G+E4.5**	**G+E1.5**	**G**	**N**	**C**
**Mice**	7.99	8.83	8.33	8.57	8.41	8.29	10.58	11.61
	7.13	7.92	12.2	8.5	9.72	9.73	12.48	10.55
	7.82	8.76	8.74	9.41	8.49	8.97	10.35	11.68

	7.02	6.91	6.99	8.95	8.43	9.85	11.48	11.46
	7.58	9.77	6.84	8.83	7.78	7.02	10.62	13.77
	7.28	9.26	10.99	7.12	8.39	9.78	11.57	10.27
	9.03	7.54	9.2	8.92	9.42	10.01	10.65	11.6
	8.52	9.55	7.61	7.39	9.55	9.35	9.33	10.43
	9.69	8.94	6.96	7.21	9.82	8.02	9.04	10.23
**Mean ± SD**	8.01 ± 0.91	8.61 ± 0.96	8.65 ± 1.89	8.32 ± 0.85	8.89 ± 0.74	9.13 ± 1.02	10.88 ± 1.08	11.29 ± 1.12
**Week 3**	**S+E4.5**	**S+E1.5**	**S**	**G+E4.5**	**G+E1.5**	**G**	**N**	**C**
	8.22	9.05	8.56	10.39	10.25	9.75	12.07	14.18
	7.23	9.23	12.77	10.25	10.38	10.63	14.19	13.93
	7.21	9.91	9.05	9.85	9.92	10.67	12.39	13.93
	7.19	7.98	7.21	9.67	10.09	11.55	13.24	14.05
**Mice**	7.86	10.19	7.42	8.02	10.98	8.93	12.99	
	7.95	10.17	11.78	9.86	10.84	11.33	12.81	13.87
	9.62	8.79	9.73	9.16	9.86	13.04	12.46	13.43
	8.71	10.45	8.45	8.89	10.87	11.84	10.63	12.49
	9.87	9.38	7.62	8.11	11.46	11.05	11.54	12.63
**Mean ± SD**	8.21 ± 1.01	9.46 ± 0.80	9.18 ± 1.95	9.36 ± 0.87	10.52 ± 0.55	10.98 ± 1.19	12.48 ± 1.02	13.56 ± 0.66
**Week 4**	**S+E4.5**	**S+E1.5**	**S**	**G+E4.5**	**G+E1.5**	**G**	**N**	**C**
	8.09	10.45	9.98	9.64	10.99	11.55	15.42	16.53
	6.47	8.64	12.28	10.94	11.73	12.96		
	7.69	9.71	10.77	10.19	10.98	11.94	13.84	17.24
	7.95	**8.75**	9.08	10.14	11.09	13.87	14.44	16.71
**Mice**	7.87	10.56	8.89	9.21	12.79	11.21	16.33	
	7.98	11.19	11.48	10.02	12.14	12.84		15.71
	8.79	9.89	9.97	9.36	10.98		14.29	15.62
	8.3	9.66	9.82	8.41	12.87	12.99	14.74	
	7.56	10.71	9.94	6.82	12.93	12.64	16.48	15.91

**Mean ± SD**	7.68 ± 0.63	9.95 ± 0.87	10.25 ± 1.09	9.41 ± 1.21	11.83 ± 0.87	12.40 ± 0.87	15.08 ± 1.03	16.29 ± 0.64
**Week 5**	**S+E4.5**	**S+E1.5**	**S**	**G+E4.5**	**G+E1.5**	**G**	**N**	**C**
	7.52	10.22	9.46	8.97	10.74	11.21	18.92	19.86
	5.27	8.47	10.27	9.1	11.69	12.52		
	7.2	9.53	10.67	9.29	10.97	11.32	17.82	
	6.96	8.61	9.36	9.12	10.54	13.15	18.94	20.48
**Mice**	7.53	10.48	9.97	8.86	13.15	11.09	18.78	
	7.39	11.03	10.38	9.51	12.85	12.62		19.95
	7.32	9.72	9.91	8.92	11.68		17.54	18.84
	7.54	9.59	9.36	8.58	12.79	12.63	18.68	
	6.12	10.64	9.85	6.7	13.04	12.37	19.85	20.18
**Mean ± SD**	6.98 ± 0.78	9.81 ± 0.88	9.91 ± 0.47	8.78 ± 0.82	11.94 ± 1.04	12.11 ± 0.79	18.65 ± 0.77	19.86 ± 0.62

SD: Standard deviation

S+E1.5: SinaAmpholeish gel (daily) with subcutaneous injection EGF 1.5 µg/kg (Alternate day)

S+E4.5: SinaAmpholeish gel (daily) with subcutaneous injection EGF 4.5 µg/kg (Alternate day)

G+E1.5: Intramuscular injection of Glucantime 20 mg/kg (daily) with subcutaneous injection EGF 1.5 µg/kg (Alternate day)

G+E4.5: Intramuscular injection of Glucantime 20 mg/kg (daily) with subcutaneous injection EGF 4.5 µg/kg (Alternate day)

G: Intramuscular injection of Glucantime 20 mg/kg (daily)

S: SinaAmpholeish gel (daily)

N: Subcutaneous injection of Normal Saline (Alternate day)

C: Control without drug treatment
