# Supplementary material for: Contribution of epidermal growth factor (EGF) in the treatment of cutaneous leishmaniasis caused by Leishmania major in BALB/c mice
Source: PLoS Negl Trop Dis. 2025 Jan 14;19(1):e0012765. doi: 10.1371/journal.pntd.0012765 (PMC11771879; doi:10.1371/journal.pntd.0012765)
Supplement: S2 Table — (PDF) [file pntd.0012765.s002.pdf]

S2 Table. Parasite burden was determined by quantitative real-time PCR on the skin lesions of BALB/c mice infected with *L. major* at the end of the fifth week of treatment.

	**qPCR (parasites/mg tissue) in BALB/c mice**				
**Groups**	**1**	**2**	**3**	**Average**	**SD**
**S+E4.5**	81791.99	78311.05	71212.81	77105.2833	5391.67574
**S+E1.5**	97549.35	96283.46	87950.94	93927.9167	5214.76816
**S**	105213.08	97204.5	92105.72	98174.4333	6607.29139
**G+E4.5**	103185.31	108996.14	98762.8	103648.083	5132.34169
**G+E1.5**	113965.23	120789.75	127114.86	120623.28	6576.3954
**G**	126148.54	113978.96	119127.65	119751.717	6108.74486
**N**	217524.25	204342.108	191703.54	204523.299	12911.3086
**C**	224870.16	219735.79	198829.31	214478.42	13793.5283

S+E1.5: SinaAmpholeish gel (daily) with subcutaneous injection EGF 1.5 µg/kg (Alternate day)

S+E4.5: SinaAmpholeish gel (daily) with subcutaneous injection EGF 4.5 µg/kg (Alternate day)

G+E1.5: Intramuscular injection of Glucantime 20 mg/kg (daily) with subcutaneous injection EGF 1.5 µg/kg (Alternate day)

G+E4.5: Intramuscular injection of Glucantime 20 mg/kg (daily) with subcutaneous injection EGF 4.5 µg/kg (Alternate day)

G: Intramuscular injection of Glucantime 20 mg/kg (daily)

S: SinaAmpholeish gel (daily)

N: Subcutaneous injection of Normal Saline (Alternate day)

C: Control without drug treatment
