# Supplementary material for: Contribution of epidermal growth factor (EGF) in the treatment of cutaneous leishmaniasis caused by Leishmania major in BALB/c mice
Source: PLoS Negl Trop Dis. 2025 Jan 14;19(1):e0012765. doi: 10.1371/journal.pntd.0012765 (PMC11771879; doi:10.1371/journal.pntd.0012765)

PLOS Neglected Tropical Diseases

Contribution of epidermal growth factor (EGF) in the treatment of cutaneous leishmaniasis caused by Leishmania major in BALB/c mice

--Manuscript Draft--

**Manuscript Number:**	PNTD-D-23-01171
**Full Title:**	Contribution of epidermal growth factor (EGF) in the treatment of cutaneous leishmaniasis caused by Leishmania major in BALB/c mice
**Short Title:**	Effect of epidermal growth factor in treatment of cutaneous leishmaniasis
**Article Type:**	Research Article
**Keywords:**	Cutaneous leishmaniasis, Epidermal growth factor, Leishmania major, Glucantime®, nano-liposomal Amphotericin B, BALB/c mice
**Abstract:**	Cutaneous leishmaniasis (CL) is a tropical disease that can leave life-long scars and lead to social stigmatization and psychological disorders. Using immunomodulatory agents that could accelerate wound healing and reduce the scar is highly demanded. Epidermal growth factor (EGF) is vital in wound healing by stimulating epidermal cell proliferation and differentiation. Here, the effect of EGF in combination with Glucantime® and nano-liposomal Amphotericin B (SinaAmpholeish®) was investigated on the healing process of CL in BALB/c mice. Seventy-two mice were infected with Leishmania major parasites and were randomly divided into eight treatment groups after the appearance of the lesion. The treatment was continued for five weeks, and lesion sizes were measured weekly. The parasite load was determined in the skin biopsies using qPCR. The wound size and parasite load were significantly lower in the SinaAmfoleish groups compared to the Glucantime ($P < 0.05$). Subcutaneous injection of EGF at 4.5 µg/kg, combined with each of the two antileishmanial drugs, significantly reduced the wound size. Using 4.5 µg/kg EGF with topical SinaAmpholeish caused a more significant reduction in lesion size and parasite load than with the intramuscular injection of Glucantime. Among the treatment groups, the EGF 4.5 µg/kg combined with SinaAmpholeish® exhibited the most significant reduction in skin diameter and parasitic load. Our results suggest that the epidermal growth factor protein can be used as a complement to anti-Leishmania drugs. Further studies are warranted to explore the beneficial effect of combining EGF with antileishmanial drugs in patients with cutaneous leishmaniasis.
**Additional Information:**	
**Question**	**Response**
**Government Employee**<br><br>Are you or any of the contributing authors an employee of the United States government?<br><br>Manuscripts authored by one or more US Government employees are not copyrighted, but are licensed under a CC0 Public Domain Dedication, which allows unlimited distribution and reuse of the article for any lawful purpose. This is a legal requirement for US Government employees.	No - No authors are employees of the U.S. government.

Powered by Editorial Manager® and ProduXion Manager® from Aries Systems Corporation

This will be typeset if the manuscript is accepted for publication.	
**Financial Disclosure**<br><br>Enter a financial disclosure statement that describes the sources of funding for the work included in this submission. Review the submission guidelines for detailed requirements. View published research articles from *PLOS Neglected Tropical Diseases* for specific examples.<br><br>This statement is required for submission and **will appear in the published article** if the submission is accepted. Please make sure it is accurate.<br><br>**Funded studies**<br>Enter a statement with the following details:<br>• Initials of the authors who received each award<br>• Grant numbers awarded to each author<br>• The full name of each funder<br>• URL of each funder website<br>• Did the sponsors or funders play any role in the study design, data collection and analysis, decision to publish, or preparation of the manuscript?<br><br>Did you receive funding for this work?	Yes
Please add funding details.<br>as follow-up to "**Financial Disclosure**<br><br>Enter a financial disclosure statement that describes the sources of funding for the work included in this submission. Review the submission guidelines for detailed requirements. View published research articles from *PLOS Neglected Tropical Diseases* for specific examples.<br><br>This statement is required for submission and **will appear in the published article** if the submission is accepted. Please make sure it is accurate.	This study was funded and supported by Tehran University of Medical Sciences (TUMS); Grant no.97.03.160.40488 and approved and supported by the Center for Research of Endemic Parasites of Iran, Tehran University of Medical Sciences, Tehran, Iran. This study was a part of the MSc thesis of Mohammad Saberi-Zow, supported by the Tehran University of Medical Sciences (grant No: 9713122001).

**Funded studies**<br>Enter a statement with the following details:<br>• Initials of the authors who received each award<br>• Grant numbers awarded to each author<br>• The full name of each funder<br>• URL of each funder website<br>• Did the sponsors or funders play any role in the study design, data collection and analysis, decision to publish, or preparation of the manuscript?<br><br>Did you receive funding for this work?"	
Please select the country of your main research funder (please select carefully as in some cases this is used in fee calculation).<br>as follow-up to "**Financial Disclosure**<br><br>Enter a financial disclosure statement that describes the sources of funding for the work included in this submission. Review the submission guidelines for detailed requirements. View published research articles from *PLOS Neglected Tropical Diseases* for specific examples.<br><br>This statement is required for submission and **will appear in the published article** if the submission is accepted. Please make sure it is accurate.<br><br>**Funded studies**<br>Enter a statement with the following details:<br>• Initials of the authors who received each award<br>• Grant numbers awarded to each author<br>• The full name of each funder<br>• URL of each funder website<br>• Did the sponsors or funders play any role in the study design, data collection and analysis, decision to publish, or preparation of the manuscript?<br><br>Did you receive funding for this work?"	IRAN, ISLAMIC REPUBLIC OF - IR
**Competing Interests**	The authors have declared that no competing interests exist.

On behalf of all authors, disclose any competing interests that could be perceived to bias this work.<br><br>This statement will be typeset if the manuscript is accepted for publication.<br><br>Review the instructions link below and PLOS NTDs' competing interests policy to determine what information must be disclosed at submission.	
**Data Availability**<br><br>Provide a **Data Availability Statement** in the box below. This statement should detail where the data used in this submission can be accessed. This statement will be typeset if the manuscript is accepted for publication.<br><br>Before publication, authors are required to make all data underlying their findings fully available, without restriction. Review our PLOS Data Policy page for detailed information on this policy. Instructions for writing your Data Availability statement can be accessed via the Instructions link below.	All relevant data are within the paper.

Manuscript
Click here to access/download;Manuscript;EGF manuscript final 9-16-2023-1.docx

Contribution of epidermal growth factor (EGF) in the treatment of cutaneous leishmaniasis caused by *Leishmania major* in BALB/c mice

Mohammad Saberi Zow[1+], Alireza Latifi[1+], Majid Golkar[2], Pezhman Fard-Esfahani[3], Sina Mohtasebi[1], Aref Teimouri[4], Mohammad Javad Abbaszadeh Afshar[5], Mehdi Mohebali[1,6*], Elham Kazemirad[1*]

[1]Department of Medical Parasitology and Mycology, School of Public Health, Tehran University of Medical Sciences, Tehran, Iran

[2]Molecular Parasitology Laboratory, Department of Parasitology, Pasteur Institute of Iran, Tehran, Iran

[3]Department of Biochemistry, Pasteur Institute of Iran, Tehran, Iran

[4]Department of Parasitology and Mycology, School of Medicine, Shiraz University of Medical Sciences, Shiraz, Iran

[5]Department of Parasitology and Mycology, School of Medicine, Jiroft University of Medical Sciences, Jiroft, Iran

[6]Center for Research of Endemic Parasites of Iran, Tehran University of Medical Sciences, Tehran, Iran

*Corresponding Authors:

E-mails: mohebali@tums.ac.ir (MM); kazemirad@tums.ac.ir, ekazemirad@yahoo.com (EK)

+These authors contributed equally to this work.

Abstract

Cutaneous leishmaniasis (CL) is a tropical disease that can leave life-long scars and lead to social stigmatization and psychological disorders. Using immunomodulatory agents that could accelerate wound healing and reduce the scar is highly demanded. Epidermal growth factor (EGF) is vital in wound healing by stimulating epidermal cell proliferation and differentiation. Here, the effect of EGF in combination with Glucantime® and nano-liposomal Amphotericin B (SinaAmpholeish®) was investigated on the healing process of CL in BALB/c mice. Seventy-two mice were infected with *Leishmania major* parasites and were randomly divided into eight treatment groups after the appearance of the lesion. The treatment was continued for five weeks, and lesion sizes were measured weekly. The parasite load was determined in the skin biopsies using qPCR. The wound size and parasite load were significantly lower in the SinaAmfoleish groups compared to the Glucantime (P <0.05). Subcutaneous injection of EGF at 4.5 µg/kg, combined with each of the two antileishmanial drugs, significantly reduced the wound size. Using 4.5 µg/kg EGF with topical SinaAmpholeish caused a more significant reduction in lesion size and parasite load than with the intramuscular injection of Glucantime. Among the treatment groups, the EGF 4.5 µg/kg combined with SinaAmpholeish® exhibited the most significant reduction in skin diameter and parasitic load. Our results suggest that the epidermal growth factor protein can be used as a complement to anti-*Leishmania* drugs. Further studies are warranted to explore the beneficial effect of combining EGF with antileishmanial drugs in patients with cutaneous leishmaniasis.

Keywords: Cutaneous leishmaniasis, Epidermal growth factor, *Leishmania major*, Glucantime®, nano-liposomal Amphotericin B, BALB/c mice

Author Summary

Cutaneous leishmaniasis (CL) is a vector-borne disease caused by *Leishmania* that affects more than 1 million people worldwide each year. CL is a disfiguring disease that can typically cause chronic skin ulcers that leave scars with significant social and economic impact. Although chemotherapy such as antimonial and amphotericin B are the pillar choice for treatment, large doses and extended treatment regimens hamper their efficacy. Using immunomodulatory agents that could accelerate the healing time and reduce the scar is highly demanded. Here, we evaluate the effectiveness of epidermal growth factor (EGF) in combination with each Glucantime® and nano-liposomal Amphotericin B (SinaAmpholeish®) in the treatment of CL in BALB/c mice. During the five-week treatment, lesion sizes were measured weekly. The parasite load was determined in the skin biopsies using qPCR. According to the result, the EGF combined with SinaAmpholeish® reduced the most significant reduction in skin diameter and parasitic load. Epidermal growth factor protein can be suggested as a supplement to anti-*Leishmania* drugs. Further studies are warranted to explore the beneficial effect of combining EGF with antileishmanial drugs in patients with cutaneous leishmaniasis.

Introduction

Leishmaniasis is a tropical disease caused by the vector-borne protozoan parasite of *Leishmania*. It is prevalent in 102 countries across five continents, with 12 to 15 million infected people, 350 million at risk, and 1.5 to 2 million new cases each year [1]. Cutaneous leishmaniasis (CL) is the most frequent clinical presentation, usually represented as a localized lesion, and often leaves disfiguring scars causing psychological, social, and economic problems [2]. Nearly 90% of CL

cases occur in Afghanistan, Algeria, Brazil, Iran, Peru, Saudi Arabia, Sudan, and Syria [1]. In the Old World, the most common etiological agents of CL are *Leishmania major* and *Leishmania tropica*, putting about 39 million people at risk [3, 4]. Iran is one of the Middle East's most major endemic countries of CL, where all clinical manifestations and epidemiological aspects of the disease have been reported in recent decades [5, 6].

Concerning the absence of an effective vaccine, chemotherapy still plays a major role in treating and controlling leishmaniasis [7]. Pentavalent antimonials, such as meglumine antimoniate (Glucantime®), have been the first-line drugs for treating all clinical forms of leishmaniasis worldwide for over 80 years [8]. However, several disadvantages are associated with this treatment, such as painful and multiple intralesional injections, long-term daily parenteral dosing, high toxicity, and treatment failure. [9, 10]. The second treatment choice is amphotericin B, which requires multiple injections with numerous side effects, variable efficacy, and drug resistance [11]. Since conventional Amphotericin B (AmB) used for intravenous injection had chronic nephrotoxic effects, liposomal formulations of AmB (AmBisome®) have been developed to reduce the toxicity and side effects [12, 13]. Intravenous (i.v.) injection of AmBisome® is mainly effective against visceral leishmaniasis, and the efficacy against cutaneous leishmaniasis is lower due to poor delivery and less accumulation within the lesion [14]. Recently, a topical formulation of nano-liposomal Amphotericin B (SinaAmpholeish 0.2%) was developed and applied to treat CL caused by *L. major* which resulted in a promising outcome in a clinical trial study [15, 16]. The nanosized liposomes as vehicles for AmB contribute to retaining the drug longer at the lesion site, enhancing drug penetration into the dermis where *Leishmania* resides, and promoting drug release [17, 18]. Although the results of topical AMB are promising in some Old World *Leishmania* species, there is a clear need to increase the cure rate, accelerate the healing time, and reduce the scar.

There is solid evidence that several factors related to the immune system and tissue regeneration promote wound healing [19]. Growth factors are critical in all stages of wound healing, including inflammation, angiogenesis, granulation tissue formation, re-epithelialization, matrix formation, and remodeling. Epidermal growth factor (EGF) is a protein secreted by platelets, macrophages, fibroblasts, and keratinocytes and is essential for dermal wound healing. It stimulates the proliferation and migration of fibroblasts and keratinocytes, the formation of granulation tissue, the contraction of wounds, and collagen deposition [20]. Also, EGF has an immunomodulatory role in inflamed skin tissue and reduces allergen-induced IL-6 production and Th17 responses in the skin [21]. Previous studies have revealed that EGF signaling promotes acute wound healing, which involves early recruitment of neutrophils, an increase in the expression of antimicrobial proteins, and, ultimately, the re-establishment of the physical barrier [22, 23]. Several studies established the effectiveness of exogenous recombinant EGF in treating active and chronic wounds, burns, and diabetic ulcers [24, 25]. Also, applying exogenous EGF to wounds can induce rapid re-epithelization, thereby reducing cutaneous scars via suppressing inflammatory reactions [26, 27]. Recently it was demonstrated that in cutaneous leishmaniasis, the expression of EGF decreased in chronic wounds, treatment failure, and relapse; however, it gradually increased in the final stages of wound healing and adequate response to chemotherapy (Montoya A 2018).

One fundamental challenge in treating CL is accelerating the healing process and preventing complications, unappealing scars, and aesthetic dissatisfaction with the patient. Since EGF could contribute to wound healing and reduce the scar, we evaluate the combined application of EGF with Glucantime and topical liposomal Amphotericin in the healing process of skin lesions caused by *Leishmania major* in BALB/c mice.

Materials and methods

Compounds

Roswell Park Memorial Institute (RPMI)-1640 medium, heat-inactivated fetal bovine serum (FBS), and the penicillin-streptomycin solution (10,000 U/mL) were purchased from Sigma-Aldrich (USA). SinaAmpholeish® (0.4% nano-liposomal Amphotericin B) was obtained from Nano Sina Exir (Iran) company. Glucantime® was purchased from Sanofi Pharmaceutical industry company (Franc). Giemsa stain was obtained from Merck chemical company (Frankfort, Germany). SinaAmpholeish (0.4% nano-liposomal amphotericin B) was obtained from Company Nano Sina Exir (Iran) and Glucantime® from Sanofi (France). The recombinant human epidermal growth factor was obtained from the Borna® Biopharma Company (Iran) and dissolved in PBS shortly before use.

Preparation of Parasite

L. major strain (MRHO/IR/75/ER) was provided by the Department of Medical Parasitology and Mycology, Tehran University of Medical Sciences, Tehran, Iran. The *Leishmania* parasite was maintained in a highly virulent state through a continuous passage in BALB/c mice. Amastigotes of *L. major* strain were isolated from the lesion of infected BALB/c mice and were transformed into promastigotes in RPMI supplemented with 10% FBS and 100 U/ml of penicillin and 100 μg/ml of streptomycin sulfate at 25±1°C. The *Leishmania* promastigotes were subcultured every four days; promastigotes from the stationary phase, between the fourth and fifth days of culture, were used to inoculate BALB/c mice.

Animals

Experiments with animals were carried out according to the ethical protocol "ARRIVE" guidelines (https://arriveguidelines.org), and measures were taken to protect animals from pain or discomfort [28]. The protocol was approved by the Tehran University of Medical Sciences ethical review board. Seventy-two male BALB/c mice, 6-8 weeks old with a body weight of approximately 20 g, were purchased from the Razi Institute of Iran, Hesarak, Karaj, Iran. All the mice were kept in a standard box and housed in a controlled animal care facility, including 23±2 ˚C temperature, 55-60% humidity, and 12 h of light-dark cycles with free access to water and a standard diet. The animals were inoculated by subcutaneous injection of 100 µl PBS containing 2×10^6 *L. major* promastigotes, from the stationary phase, into the base of the tail of mice by insulin syringe.

Ethical statements

All experimental animal procedures of this study were reviewed and approved by the Human and Animal Research Ethics Committee of the School of Public Health, Tehran University of Medical Sciences (ethical code: IR.TUMS.SPH.REC.1397.258). This study was accomplished concerning the guidelines of the Specific National Ethics for Biochemical Research issued by the Research and Technology Deputy of the Ministry of Health and Medical Education (MOHME) of Iran (issued 2005).

Treatment of infected mice

Here we evaluated two dosages of 1.5 and 4.5 µg/kg EGF subcutaneously in combination with Glucantime (20 mg $Sb5^+$/kg/day IM, 21 days) and SinaAmpholeish gel (daily, five weeks) to determine their effectiveness on lesion progression.

The treatment regimen of 1 µg/kg EGF was selected for the CL animal model as this dosage was previously shown to be effective in treating chronic wounds [29]. On the other hand, given that

the drug metabolism in animals such as rodents is faster than in humans, and also the expression of EGF is reduced in chronic lesions such as cutaneous leishmaniasis [30], a higher dose of EGF 4.5 µg/kg was also evaluated in the present study. Four weeks post-infection, the lesion was developed, and mice were selected according to lesion size to ensure similar infection levels. Then the mice were divided into eight groups, and nine were allocated to each group (Table 1). The treatment groups were as follows: C: infected, no treatment control; N: normal saline Control, 200 µl injected by subcutaneous (CS) route every other day for five weeks; G: Glucantime treatment, 20 mg $Sb5^{+}$/kg once daily for 21 days injected by intramuscular (IM) route; Groups: G+EGF1.5 and G+EGF4.5 received Glucantime treatment, (20 mg Sb^{5+}/kg/day IM, 21 days), plus EGF 1.5 µg/kg and EGF 4.5 µg/kg (200 µl), respectively, injected by subcutaneous (CS) rout every other day for five weeks; S: SinaAmpholeish gel (0.4% nano-liposomal amphotericin B), applied topically once a day for five weeks; Groups: S+EGF1.5 and S+EGF4.5 received topical SinaAmpholeish gel daily, plus EGF 1.5 µg/kg and EGF 4.5 µg/kg, respectively, injected via subcutaneous (CS) route every other day for five weeks (Table 1, Fig 1). After four weeks post-infection, when local lesions were apparent, the EGF treatment combined with the antileishmanial drug Glucantime or SinaAmpholeish was initiated for five weeks. Lesions were washed with Saline (QUIMEFA, Havana), and then 200 µl of rhEGF solution (1.5 or 4.5 µg/kg) was injected subcutaneously on alternate days using a standard disposable syringe with 27G×0.5 needles. The solution was first injected into the dermal-epidermal junction at three equidistant points all over the lesion contours and then down into the wound bottom to ensure uniform distribution. The same volume (200 µl) of normal Saline (0.02%) was also injected subcutaneously as the injection control group. Regarding antileishmanial drugs, Glucantime was administrated via Intramuscular injection

for 21 days, and topical SinaAmpholeish gel was applied once a day for five weeks alone and in combination with EGF.

After appearing the lesion and at the end of the study, samples from each lesion were prepared. Slide Smears were fixed with absolute methanol and stained with Giemsa to detect the amastigotes by the light microscope (1000×). The diameter of lesions was measured weekly before and after treatment by metric caliper.

Table 1. The treatment group's description.

Groups	Treatment	Concentration	Combination therapy	Concentration
**C**	Control group	Without treatment	**-**	**-**
**N**	Normal Saline	200 µl, 0.02% / day<br>SC injections, alternate-day (5 weeks)	-	-
**G**	Glucantime	20 mg Sb^{5+}/kg / day<br>IM injection (21 days)	-	-
**G+E1.5**	Glucantime	20 mg Sb^{5+}/kg / day<br>IM injection (21 days)	EGF	1.5 µg/kg, SC injection, alternate-day<br>(5 weeks)
**G+E4.5**	Glucantime	20 mg Sb^{5+}/kg / day<br>IM injection (21 days)	EGF	4.5 µg/kg, SC injection, alternate-day<br>(5 weeks)
**S**	SinaAmpholeish	1g nano-liposomal amphotericin B/day<br>Topical gel (5 weeks)	-	
**S+E1.5**	SinaAmpholeish	1g nano-liposomal amphotericin B/day<br>Topical gel (5 weeks)	EGF	1.5 µg/kg, SC injection, alternate-day<br>(5 weeks)
**S+E4.5**	SinaAmpholeish	1g nano-liposomal amphotericin B/day<br>Topical gel (5 weeks)	EGF	4.5 µg/kg, SC injection, alternate-day<br>(5 weeks)

Subcutaneous (SC), Intramuscular (IM), Intralesional (IL)

Evaluation of parasite burden in treated mice by quantitative real-time PCR

At the end of the treatment, three mice from each group were euthanized, and about 25–50 mg of tissue from the infected base of the tail was obtained. Genomic DNA was extracted using a High Pure PCR template preparation kit (Roche, Germany) according to the protocol provided by the manufacturer. Total genomic DNA was quantified in a NanoDrop 2000 spectrophotometer (Thermo Fischer Scientific, USA) and then used to evaluate parasite burden by quantitative real-time PCR. Each qPCR was performed in 20 µl reactions containing 40 ng target DNA, 100 nM forward and reverse primers, and 1x SYBR® Premix Ex Taq ™ II (Takara, Tokyo, Japan). Two sets of primers were used, including forward [JW11], 5′-CCTATTTTACACCAACCCCCAGT-3′ and reverse [JW12], 5′-GGGTAGGGGCGTTC TGCGAAA-3′ that amplify a 116-bp fragment of the minicircle kinetoplast DNA (kDNA) of *L. major*, present in about 10,000 copies per parasite [31]. Experiments were carried out in triplicate using a StepOnePlus Real-Time PCR System (Thermo Fisher Scientific). The PCR condition was as follows: activation at 95°C for 30 s, amplification at 95°C for 5 s, and 60°C for 30 s for 40 cycles. Finally, a melting curve analysis was performed using the following cycling parameters: 60°C for 30 sec, 5°C temperature changes to the end temperature of 95°C. The total genomic DNA of *L. major*, previously isolated from promastigotes, was serially diluted in ten-fold dilutions corresponding to 105 to 1 parasite to generate the standard curve, as described earlier [32]. A standard curve was set by plotting the Ct values against different standards with the known concentrations of the parasite's DNA. The number of parasites in the samples was calculated by interpolating the Ct of samples in the standard curve.

Histological assessment

The mice were anesthetized, and then the base of the tail with the lesion was dissected. The tissue was fixed with 4% paraformaldehyde (PFA) for 24 h at 4ºC, dehydrated in ascending ethylic

alcohols (30–100 %), cleared with 100 % xylene, and embedded in 100 % paraffin. Afterward, the samples were cut with a microtome at 5 to 10 µm thickness, stained with hematoxylin-eosin (H&E), and mounted on a glass slide for microscopic observation. In the end, morphological and pathological changes of lesion tissue were checked using light microscope images at a 200-micron scale [33].

Statistical analysis

Statistical analysis was conducted using SPSS software v.21 (IBM Analytics, USA). Statistical significances in the mean lesion size and parasite load within the groups were calculated using a one-way analysis of variance (ANOVA) test and post hoc Tukey's test (GraphPad Prism, USA *P* values < 0.05 were considered statistically significant.

Results

Effect of EGF on lesion progression in a murine model of CL

After the development of the infection, the treatment was started for five weeks, and lesion sizes were measured weekly. The mean sizes of lesions during the five weeks of different treatment regimens are presented in Table 2. The statistical analysis showed no significant difference between the lesion sizes of the eight groups in the first week. The mean size of the lesion in all the groups treated with Glucantime and SinaAmpholeish was less than that of the no-treatment control group in all the following weeks. From the second week, the mean lesion size in the Control and normal saline groups significantly increased ($p<0.05$) in comparison to the treatment groups (Table 2, Figs 1-3). In the third week of treatment, the mean wound size in SinaAmpholeish plus EGF 4.5

μg/kg (S+EGF4.5) was 8.21 ± 1.01 mm, which was significantly lower (p<0.05) than that of the normal Saline, Glucantime, and Glucantime plus EGF 1.5 μg/kg (G+EGF1.5) groups. Also, there was a significant reduction in wound size of Glucantime plus EGF 4.5 μg/kg (G+EGF4.5) and SinaAmpholeish groups compared to the Glucantime group. In the fourth week, the SinaAmpholeish plus EGF4.5 μg/kg (S+EGF4.5) group presented the lowest wound size (7.68 ± 0.63 mm) than other groups. Also, the wound size was significantly smaller in the Glucantime plus EGF4.5 μg/kg (G+EGF4.5) group compared to the Glucantime and Glucantime plus EGF 1.5 μg/kg (G+EGF1.5) groups. There was a significant reduction in lesion size in SinaAmpholeish combined with EGF1.5 μg/kg (S+EGF1.5) compared to Glucantime and Glucantime plus EGF1.5 μg/kg) (G+EGF1.5) ones.

Among the groups after 35 days, the highest mean of lesion size was reported in the not treated Control (19.86 ± 0.62 mm) and normal saline (18.65 ± 0.77 mm) groups, respectively, whereas the lowest (6.98 ± 0.78 mm) was detected in SinaAmpholeish plus EGF 4.5 μg/kg which was significant compared to other groups (P<0.05). There was no significant difference in the mean wound size between the SinaAmpholeish and SinaAmpholeish plus EGF 1.5 μg/kg groups (P> 0.05).

In the group treated with Glucantime plus EGF 4.5 μg/kg, the mean wound size was 8.78 ± 0.82 mm, significantly smaller than the Glucantime plus EGF 1.5 μg/kg and Glucantime groups (P <0.05). The lesion size did not differ significantly between the Glucantime and Glucantime plus EGF 1.5 μg/kg groups (P> 0.05) (Table 2, Figs 1-3).

On the other hand, no significant difference was observed between Glucantime plus EGF 4.5 μg/kg, SinaAmpholeish, and SinaAmpholeish plus EGF 1.5 μg/kg groups (P> 0.05).

On the whole, the SinaAmpholeish treatment groups exhibited a more significant wound size reduction than the Glucantime groups. Administration of EGF 4.5µg/kg accompanied with SinaAmpholeish, or Glucantime could significantly decrease the lesion progression.

Table 2. Comparison of mean wound size in study groups during five weeks of treatment.

The mice were divided into eight groups, and nine were allocated to each group.

Groups	Lesion size (mm) before the intervention, mean±SD	Lesion size (mm) after intervention/ Weak, mean±SD				
		1	2	3	4	5
S+E4.5	7.01±1.14	7.36±1.07	8.013±0.91*	8.21±1.01*	7.68±0.63*	6.98±0.78*
S+E1.5	7.28±1.32	7.73±1.09	8.61±0.96*	9.46±0.8*	9.95±0.87*	9.81±0.88*
S	7.02±1.94	8.04±1.85	8.65±1.89*	9.18±1.95*	10.25±1.09*	9.91±0.47*
G+E4.5	7.31±1.03	8.10±0.75	8.32±0.85*	9.36±0.87*	9.41±1.21*	8.78±0.82*
G+E1.5	7.85±1.03	8.20±0.88	8.89±0.74*	10.52±0.55*	11.83±0.87*	11.94±1.04*
G	7.33±1.84	8.10±1.5	9.13±1.02*	10.98±1.2*	12.40±0.87*	12.11±0.79*
N	8.11±1.23	8.83±0.95	10.88±1.08	12.48±1.02	15.08±1.02	18.65±0.77
C	8.10±1.27	8.81±0.7	11.29±1.12	13.56±0.65	16.29±0.64	19.86±0.62
P value vs Control	$P > 0.05$	$P > 0.05$	$P < 0.05$	$P < 0.05$	$P < 0.05$	$P < 0.05$

S + E1.5: SinaAmpholeish gel (daily) with subcutaneous injection (EGF 1.5 µg/kg) (one day in between)

S + E4.5: SinaAmpholeish gel (daily) with subcutaneous injection (EGF 4.5 µg /kg) (one day in between)

G + E1.5: Intramuscular injection of Glucantime (20 mg/kg (daily) with subcutaneous injection (EGF 1.5 µg/kg) (one day in between))

G + E4.5: Intramuscular injection of Glucantime (20 mg/kg (daily) with subcutaneous injection (EGF 4.5 µg /kg) (daily)

G: Intramuscular injection of Glucantime (20 mg/kg) (daily)

S: SinaAmpholeish gel (daily)

N: Subcutaneous injection of normal Saline (every other day)

C: Control without drug treatment

* P-value statistical analysis compared to the control group

Fig 1. In vivo efficacy of EGF 1.5 and 4.5 µg/kg combined with Glucantime® and SinaAmpholeish treatment in BALB/c mice infected with *Leishmania major*. Mice were injected (s.c.) with *L. major* promastigotes into the base of the tail of mice by insulin syringe. Animals received a five-week treatment when a nodular lesion had formed at the inoculation site. During treatment, lesion size was measured weekly. Each point represents the mean ± SEM (n = 8 per group).

Fig 2. Evaluation of lesion size in groups at the end of the fifth week. Statistical analysis was performed with One Way ANOVA, followed by the Tukey post-test, *$p < 0.05$; **$p < 0.01$; ***$p < 0.001$.

Fig 3. The lesion sizes in each group before and at the end of treatment (fifth week). After five weeks, a significant reduction in lesion size was revealed in groups treated with EGF 4.5µg/kg with liposomal Amphotericin B (SicanAmpholeish) or Glucantime.

Evaluation of parasite load in the treated BALB/c

The *L. major* load in the BALB/c was assessed using quantitative real-time PCR at the end of treatment (fifth week). As shown in Fig 4, the *L. major* parasite burden was significantly reduced in all experimental groups compared to the control groups. The group treated with SinaAmpholeish gel daily plus subcutaneous injection of EGF 4.5 µg/kg on alternate days had the lowest parasite load level. In the G+E4.5 group, the parasite load was lower than G+E1.5 and G groups. There was no significant difference between groups G and G+E1.5 and S and S+E1.5 µg /kg (Fig 4). Administration of EGF at 1.5 µg/kg concentration was ineffective in reducing parasite load.

Fig 4. **Parasite burden was determined by quantitative real-time PCR of animals infected with *L. major*.** At the end of the treatment (5th-week post-infection), the DNA was isolated from tissue of the infected base of the tail of the Balb/c. Statistical analysis was performed with One Way ANOVA, followed by the Tukey post-test, *$p < 0.05$; **$p < 0.01$; ***$p < 0.001$. Untreated, group of infected animals not treated.

Evaluation of pathologic changes

As shown in Fig 5, severe granulomatous abscess is apparent in the control group without treatment. A deep ulcer in the skin and inflammatory cells of the histiocyte/macrophage type with lymphocytic infiltration are seen in the dermis. In foci with chronic lesions, the acute inflammatory phase is seen. Amastigote can also be detected in dead macrophages in the control group where the lesion is apparent. In the group treated with nano liposomal Amphotericin B plus EGF 4.5 µg /kg, there is almost no trace of the presence of the wound, inflammatory cells, and amastigote bodies, and the wound has been repaired.

Fig 5. **Representative illustration of pathologic sections of lesions at the end of treatment (fifth week). A and B**: Severe granulomatous abscess in pathologic sections of control mice (without treatment). A deep ulcer in the skin and inflammatory cells of the histiocyte/macrophage type with lymphocytic infiltration with intracytoplasmic organisms (pseudo-amastigote) are seen in the dermis; **C and D**: Amastigotes in dead macrophages are illustrated; arrows indicate amastigotes inside parasitophorous vacuoles; **E and F**: representation of repairing tissue in the lesions treated with nano liposomal Amphotericin plus EGF 4.5 µg /kg. There is almost no trace of the presence of the wound, inflammatory cells, and amastigote bodies, and the wound has been repaired.

Discussion

Cutaneous leishmaniasis is a disfiguring disease that can leave lifelong scars and lead to social stigmatization, psychological disorders, and economic losses, especially for women and children [34]. Although chemical compounds such as antimonial and amphotericin B are the mainstay for treatment, large doses, and extended treatment regimens hinder their efficacy. Using immunomodulatory agents that could accelerate the healing time and reduce the scar is highly demanded.

Several growth factors and cytokines regulate different phases of wound healing and re-epithelialization. In this context, it was demonstrated that some growth factors were de-regulated and impaired in delayed, non-healing acute, and chronic wounds [35]. Accordingly, several

exogenous growth factors, such as EGF, have been used in the clinical setting to improve the outcomes of non-healing wounds and reduce scar [25].

EGF signaling is a crucial regulator of epithelial cell motility, which increases levels of endogenous hyaluronic acid, collagen, and elastin, thereby influencing the rate of re-epithelialization. EGF has an immunomodulatory role through the regulation of cytokine and chemokine secretion by keratinocytes and also can affect neutrophil recruitment and antimicrobial protein production [21, 22]. Choi et al. demonstrated that EGF treatment in atopic dermatitis (AD) reduced inflammatory reactions and further allergic inflammatory responses induced by Staphylococcus aureus colonization. Also, EGF increased the expression of antimicrobial proteins, β defensin-2, in human epidermal keratinocytes (HEKs), suggesting the potential antimicrobial effect of EGF [23]. Moreover, EGF reduces cutaneous scars by suppressing inflammatory reactions, decreasing the expression of TGF-b1, and mediating collagen formation [26]. It is an essential factor in healing chronic wounds as the expression of EGF decreases in the wound formation stage but increases at the final stages of wound healing [24]. In this context, several studies have established the application of topical EGF in accelerating the healing time in chronic wounds, such as venous, arterial, and diabetic ulcers, burns, and infected wounds like leprosy. Topical application of rhEGF produces skin keratinocytes and fibroblasts hyperplasia and hypertrophy, as well as corneous layer thickening and stimulates peripheral nerve regeneration. Several documents revealed that topical intralesional EGF administration could be safe and suitable to improve the therapeutic goal of healing diabetic foot ulcers. Nowadays, EGF is prepared in several nano-based systems to treat chronic wounds [36].

To date, limited studies have investigated growth factor expression in cutaneous leishmaniasis. It was revealed that in the healing stage of CL in C57BL/6 mice, Th1 cytokines and keratinocyte

growth factor (KGF) are critical in pseudo-epitheliomatous hyperplasia development and the normal healing process [37]. Also, the transforming growth factor (TGFβ) effectively stabilized cutaneous leishmaniasis infection in the early stages of wound formation and was upregulated in non-healing lesions [19]. A study searching for biomarkers of the healing process of cutaneous leishmaniasis caused by *L. braziliensis* found a differential profile of EGF, TGFβ1, PDGF, and FGF expression during the establishment of the infection and response to anti-leishmania treatment. While higher expression of TGFβ1 was more associated with acute infection and failure or relapses after treatment, the higher levels of EGF were associated with adequate wound healing [30].

Considering that CL induces long-lasting lesions and, in some cases, the healing time increased even after chemotherapy, the application of immunomodulating biomaterials such as EGF emerged as a possible complementary treatment option to drive skin tissue regeneration in CL. In this regard, for the first time, we assessed the effect of EGF in the combination of systematic Glucantime and topical nano-liposomal amphotericin B (SinaAmpholeish®) for treating CL in BALB/c mice. After a five-week treatment course, all groups treated with Glucantime and SinaAmpholeish showed remarkably smaller lesions and lower parasite load than no treatment control and normal saline groups (P <0.05). There were no significant differences between subcutaneous injection of normal Saline and no treatment control in the wound size and parasite load (P > 0.05). The mean wound size and parasite load were significantly smaller in the SinaAmfoleish groups compared to the Glucantime ones (P <0.05). One possible explanation for the better therapeutic response of nano-liposomal amphotericin B (0.04%) gel is that the concentration of the drug in the CL infection site is higher in the topical administration than in the

systemic treatment of Glucantime [38]. Also, nano-liposomes enhance drug penetration into the skin and prolong drug retention, leading to long-term sustained release at the lesion site [17, 18]. Here, two dosages of 1.5 and 4.5 µg/kg EGF were evaluated in combination with antileishmanial drugs. Administration of 1.5 µg/kg EGF, accompanied by antileishmanial treatment, did not significantly change the lesion size. On the other hand, subcutaneous injection of 4.5 µg/kg EGF, in combination with SinaAmfoleish and Glucantime, significantly reduced the wound size. However, the combination of 4.5 µg/kg EGF with topical SinaAmpholeish showed a more significant reduction in lesion size than with IM injection of Glucantime. This result could be related to the topical application of amphotericin B and the higher concentration of the drug in the lesion site [38].

Regarding parasitic load, there was a significant reduction in the treatment groups compared to the control groups. The number of parasites decreased with decreasing wound size in the treatment groups. The most significant decrease in the parasite load was found in the SinaAmfoleish plus EGF 4.5 µg/kg group. Also, in the group that received Glucantime plus EGF 4.5 µg/kg, the parasite load is slightly lower compared to Glucantime alone or with EGF 1.5 µg/kg. Combining EGF at 1.5 µg/kg with antileishmanial drugs was ineffective in reducing parasite load. Regarding pathologic changes, in the group treated with SinaAmfoleish plus EGF 4.5 µg/kg, at the end of treatment, there is almost no trace of the presence of the wound, inflammatory cells, and amastigote bodies, and the wound has been repaired.

Although the efficacy of exogenous EGF in treating leishmaniasis has not been far investigated, there is evidence of the effectiveness of other immunomodulatory factors such as granulocyte and macrophage colony-stimulating factor (GM-CSF) or mesenchymal stromal cells (AD-MSCs) on cure rate of cutaneous leishmaniasis [39, 40]. GM-CSF is a hematopoietic growth factor involved

in the proliferation and development of myeloid cells from hematopoietic stem cells. It can activate macrophages to eliminate Leishmania, enhance scar formation, and modulate the immunologic response to the lesion site. A randomized, double-blind, placebo-controlled study in Brazil demonstrated that topical administration of GM-CSF with IV injection of meglumine antimoniate reduced the healing time in CL ulcers caused by *L. braziliensis* [39]. However, the combination of topical GM-CSF with miltefosine did not improve the cure rates of CL due to *L. braziliensis* or *L. guyanensis*, which could be due to low lymphocyte proliferation, high expression of Il-10 and a T cell response toward type 2 cytokines [41, 42].

In a study by Montoya et al., the mRNA expression of growth factors was evaluated during the healing process following treatment in an animal model of cutaneous leishmaniasis caused by *L. braziliensis*. The results showed that in the active form of wound, treatment failure, and wound recurrence, TGFβ1 had the highest expression level. On the other hand, following the establishment of infection and the appearance of an ulcer, the expression level of EGF and platelet growth factor (PDEGF) drastically decreased, and with the onset of treatment and during the wound healing process, its expression gradually increased. Indeed, the inflammatory process for eliminating parasites has a negative regulatory effect on EGF expression by macrophages and fibroblasts in infected and inflamed epidermis tissue leading to the downregulation of EGF [30]. Moreover, in former studies, deficiency of EGF has been reported in chronic wounds such as diabetic foot ulcers. Also, it was reported that in chronic wounds, growth factors are trapped by the extracellular matrix or may be degraded by proteases to an excessive degree [36, 43]. According to the deficiency of EGF in cutaneous leishmaniasis, in the present study, topical administration of exogenous EGF accompanied by the antileishmanial drug could significantly reduce the wound size. Since EGF could trigger re-epithelization and wound healing and reduce

inflammatory responses in the lesion site, it can be suggested as a promising supplement for treating CL in which it was downregulated. It is worth mentioning that different nanoparticles loaded with recombinant EGF are available in marketing to protect EGF from enzymatic degradation in the proteolytic wound environment. Also, topical nanomaterials EGF gel, cream, or spray were used in several animal models and clinical trials, which is more feasible and painless than intralesional or subcutaneous administration [36]. Using the topical nano-based delivery system of EGF plus topical antileishmanial drugs such as nano-liposomal amphotericin B could be a promising tool to accelerate wound healing of CL in the future.

Conclusion

Overall, here for the first time, the effect of EGF in combination with an antileishmanial drug was investigated for treating CL. Our results demonstrated that administration of EGF 4.5 µg/kg, in combination with SinaAmfoleish or Glucantime, significantly decreased the wound size. However, the combination of 4.5 µg/kg EGF with topical SinaAmpholeish showed the most significant reduction in lesion size and parasite load. Among the groups, the topical administration of SinaAmfoleish has a more therapeutic effect than the intramuscular injection of Glucantime. Further studies have been suggested to evaluate EGF and topical nano-based EGF in combination with the antileishmanial drug in the animal model and clinical trial, which could be a promising tool for the treatment.

Funding

This study was funded and supported by Tehran University of Medical Sciences (TUMS); Grant no.97.03.160.40488 and approved and supported by the Center for Research of Endemic Parasites of Iran, Tehran University of Medical Sciences, Tehran, Iran. This study was a part of the MSc

thesis of Mohammad Saberi-Zow, supported by the Tehran University of Medical Sciences (grant No: 9713122001).

Acknowledgments

The authors would like to acknowledge all colleagues of the Borna® Biopharma Company, for assistance in preparing recombinant epidermal growth factor. We thank Dr. Ahad Mohammadnejad for helping with the histopathological assessment.

References

1. Alvar J, Vélez ID, Bern C, Herrero M, Desjeux P, Cano J, et al. Leishmaniasis worldwide and global estimates of its incidence. PloS one. 2012;7(5):e35671. Epub 2012/06/14. doi: 10.1371/journal.pone.0035671. PubMed PMID: 22693548; PubMed Central PMCID: PMCPMC3365071.
2. Reithinger R, Dujardin JC, Louzir H, Pirmez C, Alexander B, Brooker S. Cutaneous leishmaniasis. The Lancet Infectious diseases. 2007;7(9):581-96. Epub 2007/08/24. doi: 10.1016/s1473-3099(07)70209-8. PubMed PMID: 17714672.
3. Salam N, Al-Shaqha WM, Azzi A. Leishmaniasis in the Middle East: Incidence and Epidemiology. PLOS Neglected Tropical Diseases. 2014;8(10):e3208. doi: 10.1371/journal.pntd.0003208.
4. Ghatee MA, Taylor WR, Karamian M. The Geographical Distribution of Cutaneous Leishmaniasis Causative Agents in Iran and Its Neighboring Countries, A Review. Frontiers in public health. 2020;8:11. Epub 2020/03/07. doi: 10.3389/fpubh.2020.00011. PubMed PMID: 32133334; PubMed Central PMCID: PMCPMC7039857.
5. Norouzinezhad F, Ghaffari F, Norouzinejad A, Kaveh F, Gouya MM. Cutaneous leishmaniasis in Iran: Results from an epidemiological study in urban and rural provinces. Asian Pacific Journal of Tropical Biomedicine. 2016;6(7):614-9. doi: https://doi.org/10.1016/j.apjtb.2016.05.005.
6. Postigo JA. Leishmaniasis in the World Health Organization Eastern Mediterranean Region. International journal of antimicrobial agents. 2010;36 Suppl 1:S62-5. Epub 2010/08/24. doi: 10.1016/j.ijantimicag.2010.06.023. PubMed PMID: 20728317.
7. Kedzierski L, Sakthianandeswaren A, Curtis JM, Andrews PC, Junk PC, Kedzierska K. Leishmaniasis: current treatment and prospects for new drugs and vaccines. Current medicinal chemistry. 2009;16(5):599-614. Epub 2009/02/10. doi: 10.2174/092986709787458489. PubMed PMID: 19199925.
8. Croft SL, Olliaro P. Leishmaniasis chemotherapy--challenges and opportunities. Clinical microbiology and infection : the official publication of the European Society of Clinical

Microbiology and Infectious Diseases. 2011;17(10):1478-83. Epub 2011/09/22. doi: 10.1111/j.1469-0691.2011.03630.x. PubMed PMID: 21933306.
9. Sundar S, Chakravarty J. Antimony toxicity. International journal of environmental research and public health. 2010;7(12):4267-77. Epub 2011/02/15. doi: 10.3390/ijerph7124267. PubMed PMID: 21318007; PubMed Central PMCID: PMCPMC3037053.
10. Mohebali M, Kazemirad E, Hajjaran H, Kazemirad E, Oshaghi MA, Raoofian R, et al. Gene expression analysis of antimony resistance in Leishmania tropica using quantitative real-time PCR focused on genes involved in trypanothione metabolism and drug transport. Archives of Dermatological Research. 2019;311(1):9-17. doi: 10.1007/s00403-018-1872-2.
11. Uliana SRB, Trinconi CT, Coelho AC. Chemotherapy of leishmaniasis: present challenges. Parasitology. 2018;145(4):464-80. Epub 2017/01/20. doi: 10.1017/S0031182016002523.
12. Adler-Moore JP, Gangneux JP, Pappas PG. Comparison between liposomal formulations of amphotericin B. Medical mycology. 2016;54(3):223-31. Epub 2016/01/16. doi: 10.1093/mmy/myv111. PubMed PMID: 26768369.
13. Wijnant GJ, Van Bocxlaer K, Yardley V, Harris A, Alavijeh M, Silva-Pedrosa R, et al. Comparative efficacy, toxicity and biodistribution of the liposomal amphotericin B formulations Fungisome(®) and AmBisome(®) in murine cutaneous leishmaniasis. International journal for parasitology Drugs and drug resistance. 2018;8(2):223-8. Epub 2018/04/21. doi: 10.1016/j.ijpddr.2018.04.001. PubMed PMID: 29673889; PubMed Central PMCID: PMCPMC6039306.
14. Iman M, Huang Z, Alavizadeh SH, Szoka FC, Jr., Jaafari MR. Biodistribution and In Vivo Antileishmanial Activity of 1,2-Distigmasterylhemisuccinoyl-sn-Glycero-3-Phosphocholine Liposome-Intercalated Amphotericin B. Antimicrobial agents and chemotherapy. 2017;61(9). Epub 2017/06/21. doi: 10.1128/aac.02525-16. PubMed PMID: 28630182; PubMed Central PMCID: PMCPMC5571372.
15. Eskandari SE, Firooz A, Nassiri-Kashani M, Jaafari MR, Javadi A, Miramin Mohammadi A, et al. Safety Evaluation of Topical Application of Nano-Liposomal Form of Amphotericin B (SinaAmpholeish) on Healthy Volunteers: Phase I Clinical Trial. Iranian journal of parasitology. 2019;14(2):197-203. Epub 2019/09/24. PubMed PMID: 31543907; PubMed Central PMCID: PMCPMC6737359.
16. Jaafari MR, Hatamipour M, Alavizadeh SH, Abbasi A, Saberi Z, Rafati S, et al. Development of a topical liposomal formulation of Amphotericin B for the treatment of cutaneous leishmaniasis. International journal for parasitology Drugs and drug resistance. 2019;11:156-65. Epub 2019/10/05. doi: 10.1016/j.ijpddr.2019.09.004. PubMed PMID: 31582344; PubMed Central PMCID: PMCPMC6904837.
17. El Maghraby GM, Barry BW, Williams AC. Liposomes and skin: from drug delivery to model membranes. European journal of pharmaceutical sciences : official journal of the European Federation for Pharmaceutical Sciences. 2008;34(4-5):203-22. Epub 2008/06/24. doi: 10.1016/j.ejps.2008.05.002. PubMed PMID: 18572392.
18. Perez AP, Altube MJ, Schilrreff P, Apezteguia G, Celes FS, Zacchino S, et al. Topical amphotericin B in ultradeformable liposomes: Formulation, skin penetration study, antifungal and antileishmanial activity in vitro. Colloids and surfaces B, Biointerfaces. 2016;139:190-8. Epub 2015/12/29. doi: 10.1016/j.colsurfb.2015.12.003. PubMed PMID: 26709977.
19. Abdoli A, Maspi N, Ghaffarifar F. Wound healing in cutaneous leishmaniasis: A double edged sword of IL-10 and TGF-β. Comparative immunology, microbiology and infectious

diseases. 2017;51:15-26. Epub 2017/05/16. doi: 10.1016/j.cimid.2017.02.001. PubMed PMID: 28504090.

20. Singla S, Singla S, Kumar A, Singla M. Role of epidermal growth factor in healing of diabetic foot ulcers. The Indian journal of surgery. 2012;74(6):451-5. Epub 2013/12/03. doi: 10.1007/s12262-012-0447-2. PubMed PMID: 24293898; PubMed Central PMCID: PMCPMC3537988.

21. Zhang Z, Xiao C, Gibson AM, Bass SA, Khurana Hershey GK. EGFR signaling blunts allergen-induced IL-6 production and Th17 responses in the skin and attenuates development and relapse of atopic dermatitis. Journal of immunology (Baltimore, Md : 1950). 2014;192(3):859-66. Epub 2013/12/18. doi: 10.4049/jimmunol.1301062. PubMed PMID: 24337738; PubMed Central PMCID: PMCPMC3946981.

22. Pastore S, Mascia F. Novel acquisitions on the immunoprotective roles of the EGF receptor in the skin. Expert review of dermatology. 2008;3(5):525-7. Epub 2009/08/04. doi: 10.1586/17469872.3.5.525. PubMed PMID: 19649143; PubMed Central PMCID: PMCPMC2600505.

23. Choi SY, Lee YJ, Kim JM, Kang HJ, Cho SH, Chang SE. Epidermal Growth Factor Relieves Inflammatory Signals in Staphylococcus aureus-Treated Human Epidermal Keratinocytes and Atopic Dermatitis-Like Skin Lesions in Nc/Nga Mice. BioMed research international. 2018;2018:9439182. Epub 2018/06/05. doi: 10.1155/2018/9439182. PubMed PMID: 29862299; PubMed Central PMCID: PMCPMC5976919.

24. Choi JK, Jang JH, Jang WH, Kim J, Bae IH, Bae J, et al. The effect of epidermal growth factor (EGF) conjugated with low-molecular-weight protamine (LMWP) on wound healing of the skin. Biomaterials. 2012;33(33):8579-90. Epub 2012/08/23. doi: 10.1016/j.biomaterials.2012.07.061. PubMed PMID: 22910220.

25. Fernández-Montequín JI, Betancourt BY, Leyva-Gonzalez G, Mola EL, Galán-Naranjo K, Ramírez-Navas M, et al. Intralesional administration of epidermal growth factor-based formulation (Heberprot-P) in chronic diabetic foot ulcer: treatment up to complete wound closure. International wound journal. 2009;6(1):67-72. Epub 2009/03/18. doi: 10.1111/j.1742-481X.2008.00561.x. PubMed PMID: 19291119; PubMed Central PMCID: PMCPMC7951202.

26. Kim YS, Lew DH, Tark KC, Rah DK, Hong JP. Effect of recombinant human epidermal growth factor against cutaneous scar formation in murine full-thickness wound healing. Journal of Korean medical science. 2010;25(4):589-96. Epub 2010/04/02. doi: 10.3346/jkms.2010.25.4.589. PubMed PMID: 20358003; PubMed Central PMCID: PMCPMC2844601.

27. Shin SH, Koh YG, Lee WG, Seok J, Park KY. The use of epidermal growth factor in dermatological practice. International wound journal. 2022. Epub 2022/12/31. doi: 10.1111/iwj.14075. PubMed PMID: 36584669.

28. Percie du Sert N, Ahluwalia A, Alam S, Avey MT, Baker M, Browne WJ, et al. Reporting animal research: Explanation and elaboration for the ARRIVE guidelines 2.0. PLOS Biology. 2020;18(7):e3000411. doi: 10.1371/journal.pbio.3000411.

29. Ryu SH, Moon SY, Yang YJ, Moon SR, Hong JP, Choi J, et al. Recombinant human epidermal growth factor accelerates the proliferation of irradiated human fibroblasts and keratinocytes in vitro and in vivo. Journal of radiation research. 2009;50(6):545-52. Epub 2009/12/05. doi: 10.1269/jrr.09066. PubMed PMID: 19959882.

30. Montoya A, Yepes L, Bedoya A, Henao R, Delgado G, Vélez ID, et al. Transforming Growth Factor Beta (TGFβ1) and Epidermal Growth Factor (EGF) as Biomarkers of Leishmania (V) braziliensis Infection and Early Therapeutic Response in Cutaneous Leishmaniasis: Studies in

Hamsters. Frontiers in cellular and infection microbiology. 2018;8:350. Epub 2018/10/20. doi: 10.3389/fcimb.2018.00350. PubMed PMID: 30333964; PubMed Central PMCID: PMCPMC6176012.

31. Nicolas L, Sidjanski S, Colle JH, Milon G. Leishmania major reaches distant cutaneous sites where it persists transiently while persisting durably in the primary dermal site and its draining lymph node: a study with laboratory mice. Infection and immunity. 2000;68(12):6561-6. Epub 2000/11/18. doi: 10.1128/iai.68.12.6561-6566.2000. PubMed PMID: 11083765; PubMed Central PMCID: PMCPMC97750.

32. Heidari-Kharaji M, Taheri T, Doroud D, Habibzadeh S, Rafati S. Solid lipid nanoparticle loaded with paromomycin: in vivo efficacy against Leishmania tropica infection in BALB/c mice model. Applied Microbiology and Biotechnology. 2016;100(16):7051-60. doi: 10.1007/s00253-016-7422-y.

33. Haghshenas M, Koosha M, Latifi A, Kazemirad E, Dehghan A, Nikmanesh B, et al. Detection of Enterobius vermicularis in archived formalin-fixed paraffin-embedded (FFPE) appendectomy blocks: It's potential to compare genetic variations based on mitochondrial DNA (cox1) gene. PloS one. 2023;18(2):e0281622. Epub 2023/02/10. doi: 10.1371/journal.pone.0281622. PubMed PMID: 36758053; PubMed Central PMCID: PMCPMC9910638.

34. Bilgic-Temel A, Murrell DF, Uzun S. Cutaneous leishmaniasis: A neglected disfiguring disease for women. International journal of women's dermatology. 2019;5(3):158-65. Epub 2019/07/31. doi: 10.1016/j.ijwd.2019.01.002. PubMed PMID: 31360749; PubMed Central PMCID: PMCPMC6637076.

35. Yamakawa S, Hayashida K. Advances in surgical applications of growth factors for wound healing. Burns & trauma. 2019;7:10. Epub 2019/04/18. doi: 10.1186/s41038-019-0148-1. PubMed PMID: 30993143; PubMed Central PMCID: PMCPMC6450003.

36. Park JW, Hwang SR, Yoon IS. Advanced Growth Factor Delivery Systems in Wound Management and Skin Regeneration. Molecules (Basel, Switzerland). 2017;22(8). Epub 2017/07/28. doi: 10.3390/molecules22081259. PubMed PMID: 28749427; PubMed Central PMCID: PMCPMC6152378.

37. Akilov OE, Donovan MJ, Stepinac T, Carter CR, Whitcomb JP, Hasan T, et al. T helper type 1 cytokines and keratinocyte growth factor play a critical role in pseudoepitheliomatous hyperplasia initiation during cutaneous leishmaniasis. Arch Dermatol Res. 2007;299(7):315-25. Epub 2007/07/24. doi: 10.1007/s00403-007-0765-6. PubMed PMID: 17643254.

38. Tareen A, Ahmad SA, Khan I-u, editors. Comparison of the efficacy of intralesional versus intramuscular injection Meglumine Antimoniate in the treatment of sores of Cutaneous Leishmaniasis2016.

39. Santos JB, de Jesus AR, Machado PR, Magalhães A, Salgado K, Carvalho EM, et al. Antimony plus recombinant human granulocyte-macrophage colony-stimulating factor applied topically in low doses enhances healing of cutaneous Leishmaniasis ulcers: a randomized, double-blind, placebo-controlled study. The Journal of infectious diseases. 2004;190(10):1793-6. Epub 2004/10/23. doi: 10.1086/424848. PubMed PMID: 15499535.

40. Ramos TD, Silva JD, da Fonseca-Martins AM, da Silveira Pratti JE, Firmino-Cruz L, Maciel-Oliveira D, et al. Combined therapy with adipose tissue-derived mesenchymal stromal cells and meglumine antimoniate controls lesion development and parasite load in murine cutaneous leishmaniasis caused by Leishmania amazonensis. Stem cell research & therapy.

2020;11(1):374. Epub 2020/09/02. doi: 10.1186/s13287-020-01889-z. PubMed PMID: 32867857; PubMed Central PMCID: PMCPMC7457509.

41. Machado PRL, Prates FVO, Boaventura V, Lago T, Guimarães LH, Schriefer A, et al. A Double-blind, Randomized Trial to Evaluate Miltefosine and Topical Granulocyte Macrophage Colony-stimulating Factor in the Treatment of Cutaneous Leishmaniasis Caused by Leishmania braziliensis in Brazil. Clinical infectious diseases : an official publication of the Infectious Diseases Society of America. 2021;73(7):e2465-e9. Epub 2020/09/08. doi: 10.1093/cid/ciaa1337. PubMed PMID: 32894278; PubMed Central PMCID: PMCPMC8492114.

42. Mendes L, Guerra JO, Costa B, Silva ASD, Guerra M, Ortiz J, et al. Association of miltefosine with granulocyte and macrophage colony-stimulating factor (GM-CSF) in the treatment of cutaneous leishmaniasis in the Amazon region: A randomized and controlled trial. International journal of infectious diseases : IJID : official publication of the International Society for Infectious Diseases. 2021;103:358-63. Epub 2020/12/01. doi: 10.1016/j.ijid.2020.11.183. PubMed PMID: 33253864.

43. Blakytny R, Jude E. The molecular biology of chronic wounds and delayed healing in diabetes. Diabetic medicine : a journal of the British Diabetic Association. 2006;23(6):594-608. Epub 2006/06/09. doi: 10.1111/j.1464-5491.2006.01773.x. PubMed PMID: 16759300.

Figure 1

Click here to access/download;Figure;Fig 1.tif

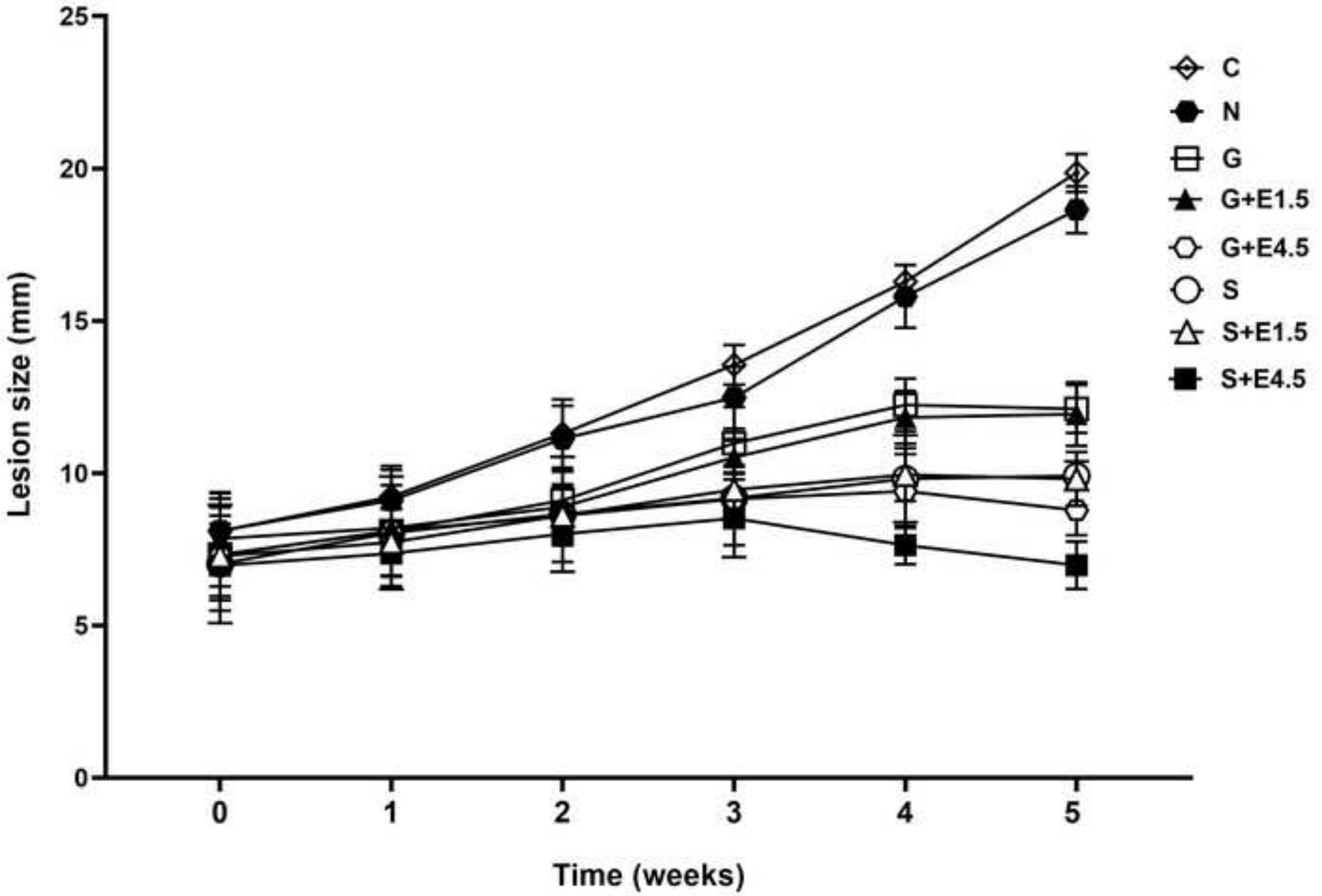

Figure 2

Click here to access/download;Figure;Fig 2.tif

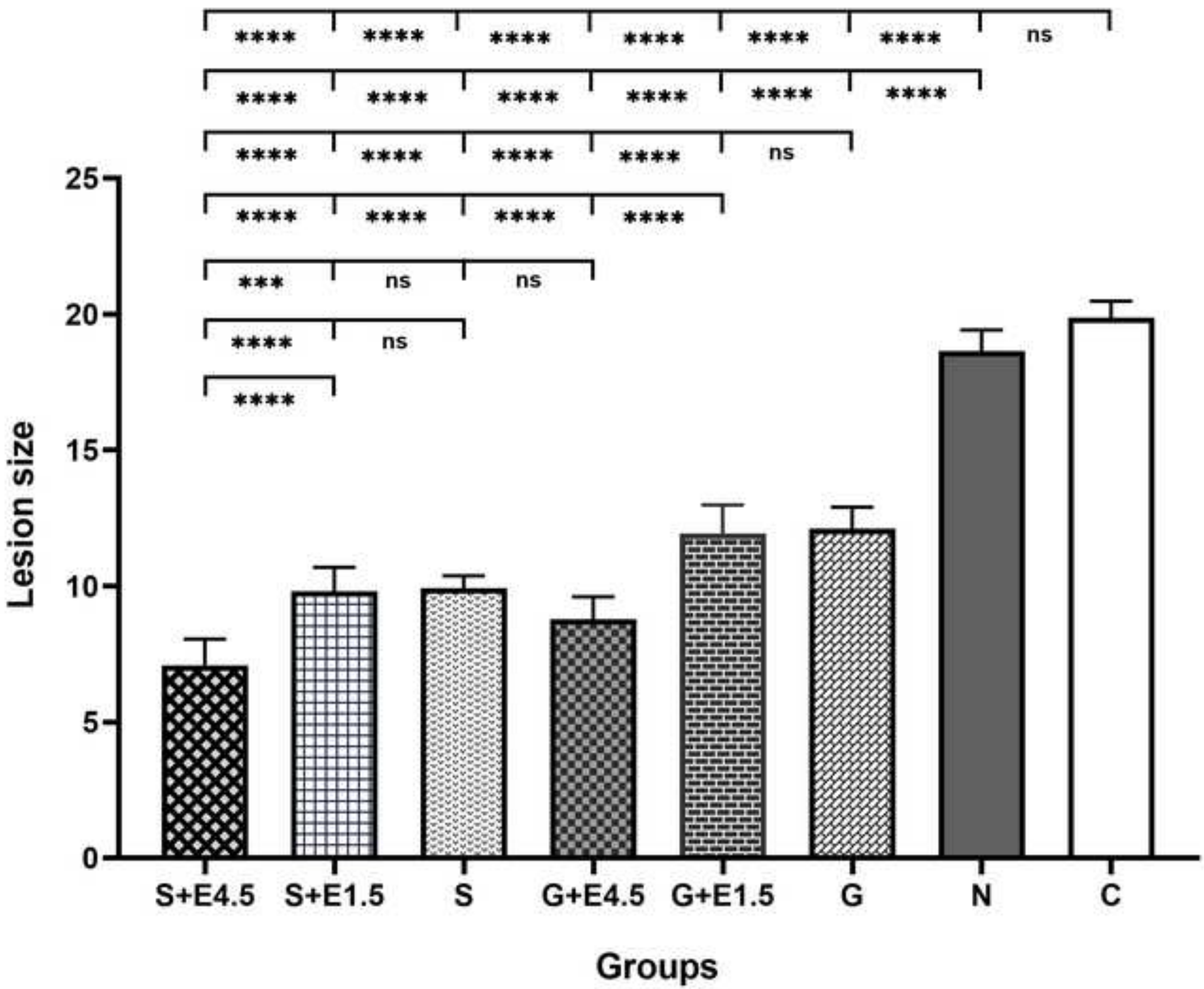

Figure 3

Click here to access/download;Figure;Fig 3.jpg

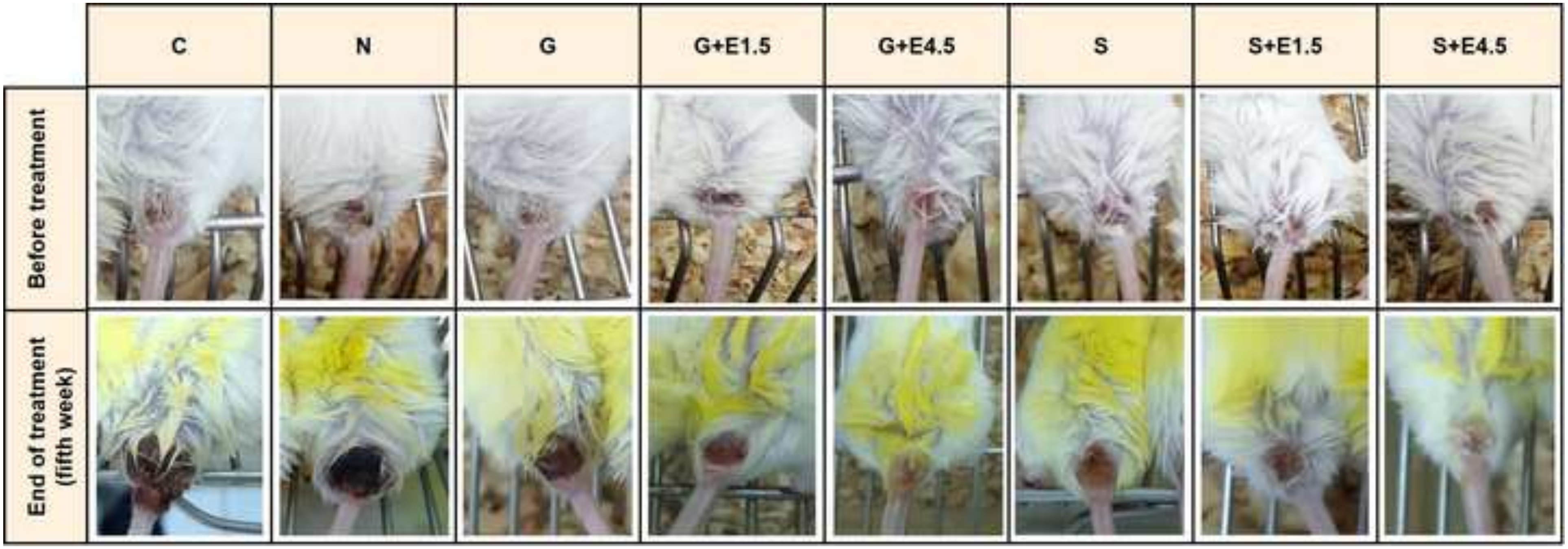

Figure 4

Click here to access/download;Figure;Fig 4.tif

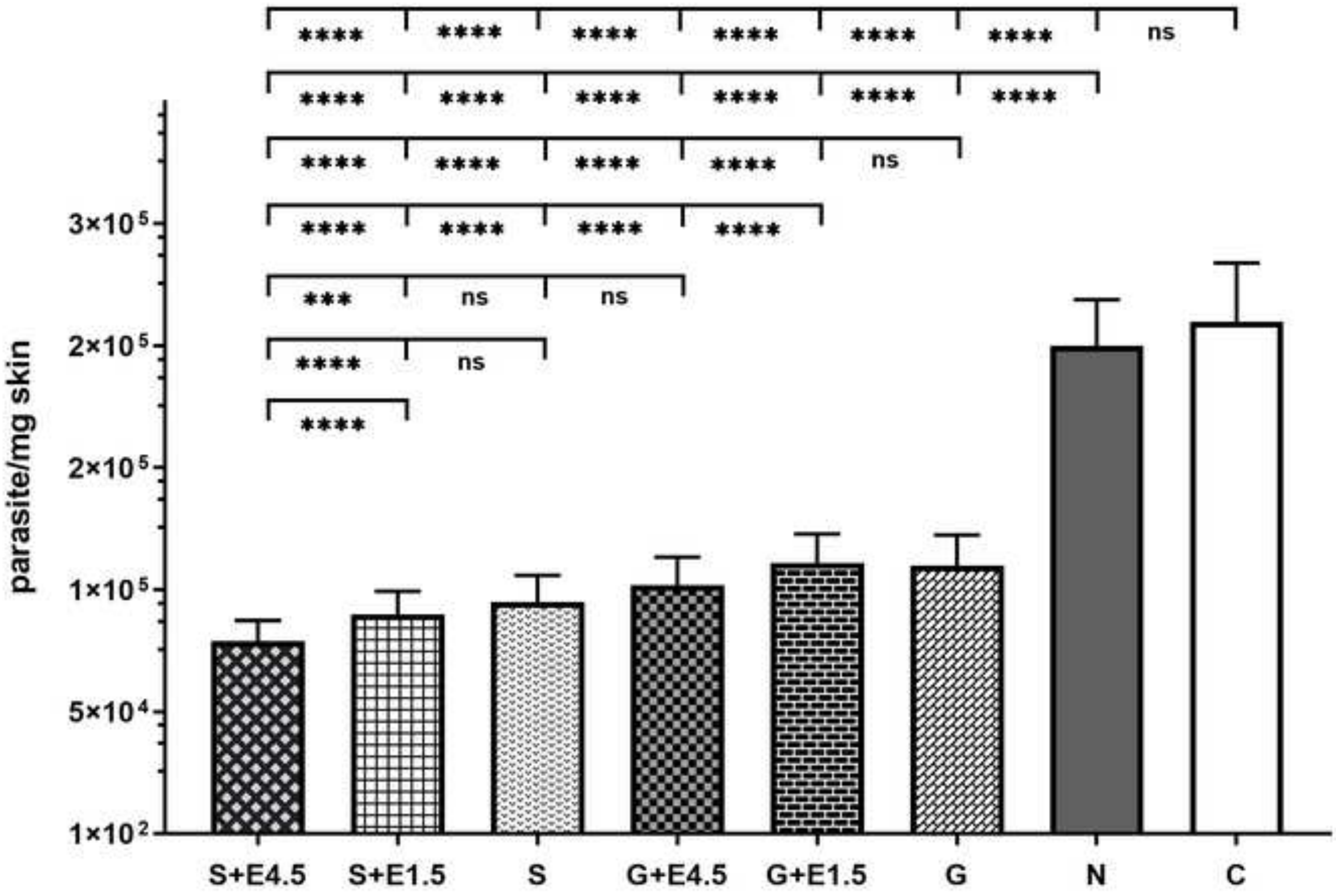

Figure 5

Click here to access/download;Figure;Fig 5.jpg

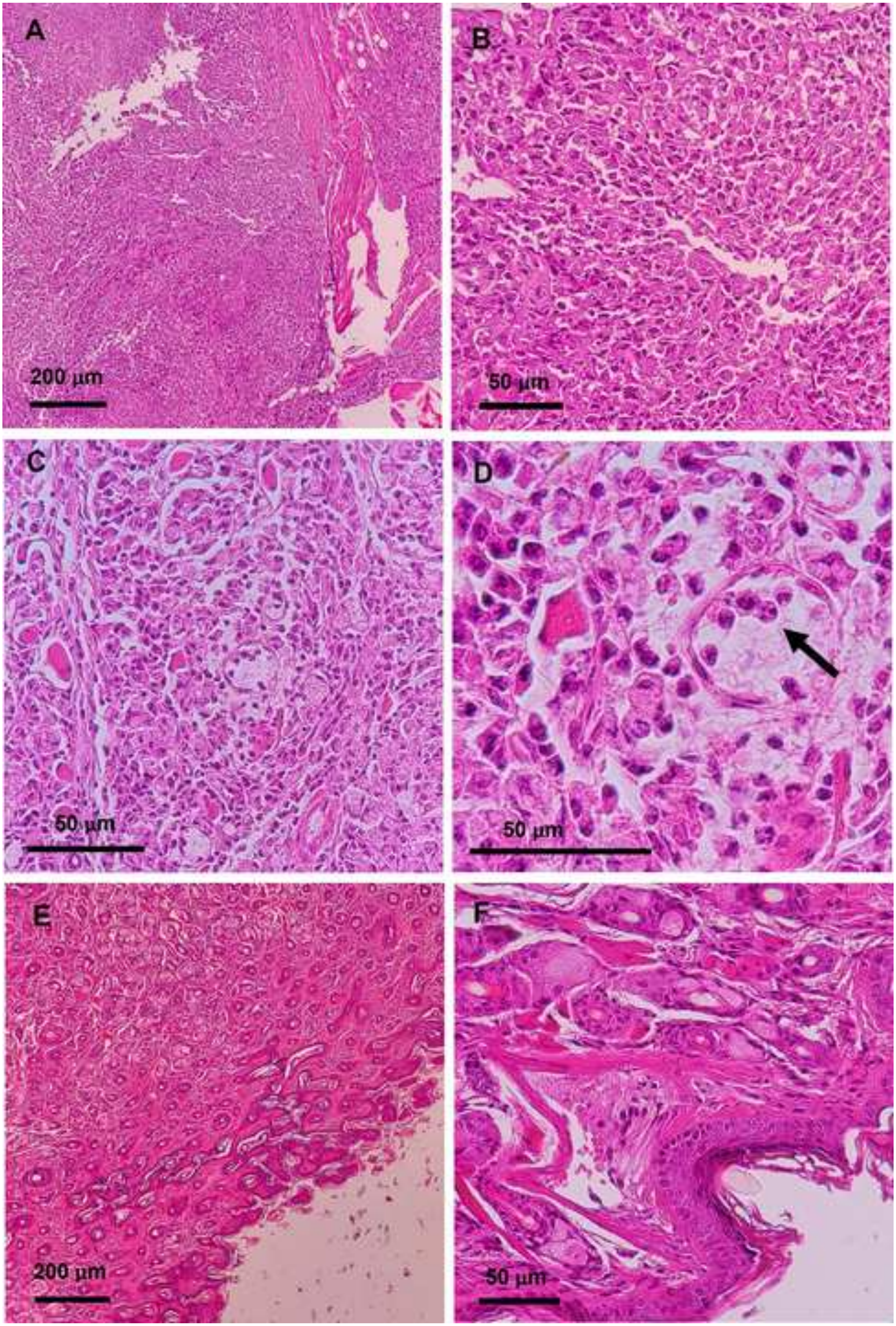

Supplement: Attachment — Submitted filename: PNTD-D-23-01171_reviewer.pdf [file pntd.0012765.s003.pdf]
