# Supplementary material for: Contribution of epidermal growth factor (EGF) in the treatment of cutaneous leishmaniasis caused by Leishmania major in BALB/c mice
Source: PLoS Negl Trop Dis. 2025 Jan 14;19(1):e0012765. doi: 10.1371/journal.pntd.0012765 (PMC11771879; doi:10.1371/journal.pntd.0012765)
Supplement: Attachment — Submitted filename: Respond to reviewer.docx [file pntd.0012765.s004.docx]

**Dear Prof. Claudia Ida Brodskyn**

**Editor,**

**PLOS Neglected Tropical Diseases	30-April-2024**

First, I would like to thank you and the Editorial Office for solving the technical problem with my ORCID ID and extending the due date.

Regarding your email concerning our manuscript entitled: "**Contribution of epidermal growth factor (EGF) in the treatment of cutaneous leishmaniasis caused by *Leishmania major* in BALB/c mice**" I would like to thank you for your informative email and the reviewers for their valuable and critical comments. I would like to inform you that the manuscript has been entirely edited, and all the following corrections and changes were made based on reviewers' suggestions. New and increased subjects were highlighted in the manuscript.

We hope the changes listed below meet the publication requirements.

I sincerely appreciate your consideration and look forward to hearing from you.

Sincerely Yours,

Mehdi Mohebali,

Corresponding author, Professor of Medical Parasitology

Department of Parasitology & Mycology, Tehran University of Medical Sciences,

Tel/Fax: +9821-88951392

**Reviewer #1: The authors test the effect of EGF in combination with anti-leishmanial therapy on wound healing and parasite load. The sample size used to measure lesion size for the many groups tested is reasonable. However, in the analysis of parasite load only 3 mice per group are included according to the information given in the methods sections. This number is not sufficient.**

***Response*:** Thank you for raising this point. The minimum sample size for statistical analysis of parasite load by qPCR is three, though as the sample size increases, so does the power of the significance test. However, in some studies, this sample size was used for analyzing parasite load (Gonçalves-Oliveira et al., 2019; Jaafari et al., 2019; Nicolas et al., 2000). According to the previous reports, we conducted the quantitative real-time PCR on three randomly selected animals in each group. Moreover, the qPCR method is a validated and reliable method routinely used for diagnosing leishmaniasis in patients in our laboratory with consensus results. The references were added to the M&M to support our data (line 197, highlighted in red).

Moreover, as stated in the M&M, before starting the treatment and at the end of the fifth week, samples from each lesion were prepared and stained with Giemsa 10% to detect the amastigotes by the light microscope. According to the previous studies, parasite loads in the skin lesion also had determined based on the WHO guidelines in all the groups using microscopy as ordinal division, including grade 0 (0 parasites in 1000 fields), grade 1+ (1–10 parasites in 1000 fields), grade 2+ (1–10 parasites in 100 fields), grade 3+ (1–10 parasites in 10 fields), grade 4+ (1–10 parasites per field), grade 5+ (10–100 parasites per field), or grade 6+ (> 100 parasites per field) (Ghosh et al., 2022; Mohtasebi et al., 2019; Pouryousef et al., 2021; WHO, 2010). The microscopy results revealed that the parasite load was significantly reduced in the groups that received antileishmanial drugs compared to the untreated control group (*p*< 0.05). The group receiving EGF 4.5 µg/kg plus amphotericin B displayed the lowest amastigote number in the smears. However, since in microscopical grading, the distances among the numbers of amastigotes are wide, and probability errors for their count are high, we evaluated the parasite load via qPCR on three randomly selected animals in each group. Given that the results of parasite load by qPCR agreed with the reduction of lesion size in the groups administrated EGF 4.5 µg/mL supplement, we did not include the microscopy results in the manuscript.

**Moreover, a group only treated with the high dose EFG alone would have been relevant to include since the effect of this growth factor on Leishmania parasite growth is not known and with EFG having effects on both lesion size and potentially parasite load.**

***Response:***

EGF is a growth factor that is used in topical or intralesional forms for the healing of chronic wounds. Several clinical trials demonstrated that EGF is not pharmacologically considered an antimicrobial agent, and its main function is to prompt wound healing (Park et al., 2017; Shin et al., 2023). Therefore, nowadays, combination therapy of EGF and antibiotics are extensively used to treat and accelerate the healing of infected wounds in clinical settings. In this context, clinical trials demonstrated that using these two reagents simultaneously, EGF significantly promotes wound healing, and silver or silver sulfadiazine can effectively prevent infection. Therefore, they jointly contribute to wound repair and shorten the healing time compared to EGF alone (Lee et al., 2021; Li et al., 2021; Zhang et al., 2021). Recently, in a self-controlled clinical trial, autologous platelet gel (APG) prepared from platelet-rich plasma (PRP) containing natural growth factors in combination with Glucantime was used in a limited number of CL patients (n=13). The combination therapy led to accelerated wound healing compared to monotherapy with Glucanime; however, the effect of APG alone was not evaluated (Shadmand et al., 2023). This was summarized and mentioned in the limitation, highlighted in yellow.

Several studies revealed that chronic refractory wounds have low levels of growth factors such as EGF; consequently, applying exogenous EGF could compensate for the diminished EGF levels and promote wound healing (Park et al., 2017). An interesting study (Montoya et al., 2018) showed that EGF expression decreased in the lesions of cutaneous leishmaniasis. As such, we aimed to test the hypothesis that the addition of EGF to the anti-*Leishmania* therapy would have a synergistic effect in the treatment of cutaneous leishmaniasis. Our results clearly showed that the simultaneous application of EGF and antileishmanial drugs improved the healing of cutaneous leishmaniasis. Interestingly, groups receiving anti-*Leishmania* drugs plus 4.5 µg/kg of EGF had lesions significantly smaller compared to the control groups receiving only Glucantime or Amphotericin B. Indeed, the antileishmanial drugs inhibit parasite growth, and exogenous EGF probably compensates for the growth factor deficiency in the CL lesion, responsible for accelerating re-[epithelialization](https://www.google.com/search?sca_esv=46b4c7e13b37a354&sca_upv=1&sxsrf=ACQVn0-xplPNk0Y1d0A4zdESs8qJ66AjDw:1712909913498&q=epithelialization&spell=1&sa=X&ved=2ahUKEwislLTXnryFAxXX9gIHHXlqBnUQkeECKAB6BAgHEAI) of the wound, and synergistically contribute to the promoting wound healing. The efficacy of EGF in healing *Leishmania* lesions was probably due to accelerating re-epithelization and reducing inflammatory responses in the wound. We argued these statements in the discussion section and also in the limitation (underlined in the text). Also, in the limitation of the study, we mentioned not including the EGF group. We suggested further analysis could be helpful regarding the effect of exogenous EGF alone on the wound healing process, the host inflammation response, and scar formation in cutaneous leishmaniasis (highlighted in yellow).

**The source of EFG should be included in the methods.**

***Response:*** In the present study, the human recombinant epidermal growth factor (rhEGF) was prepared from the Borna Biopharma (Iran) and dissolved in PBS shortly before use; it was stated in the Compound in the M&M (highlighted in dark yellow).

--------------------

**No concerns regarding ethics or the statistical method selected.**

**Reviewer #2:** Are the objectives of the study clearly articulated with a clear testable hypothesis stated? Yes.

-Is the study design appropriate to address the stated objectives? Yes.

-Is the population clearly described and appropriate for the hypothesis being tested? Yes.

-Is the sample size sufficient to ensure adequate power to address the hypothesis being tested? Yes

-Were correct statistical analysis used to support conclusions? Yes

-Are there concerns about ethical or regulatory requirements being met? No.

**Reviewer #3: - The objective is clear but the methods and the results are not very clear**

***Response:*** We edited the method and result and clarified them in detail.

**- Cutaneous leishmaniasis due to L. major is associated with a self-cure rate above 50%–75% at 4–6 months. So it is not clear how this was assessed.**

***Response:***

Susceptibility and resistance to *Leishmania* infection in humans and mice are broadly varied and regulated by genetic determinants (Blackwell et al., 2009). Usually, *L. major* is self-healing in humans, and after 4-6 months, it recovers. However, several strains of mice, such as BALB/c, SWR/J, and P/J are susceptible to different *Leishmania* species and develop severe non-healing systemic disease affecting the skin and visceral organs (Kurey et al., 2009; Sacks and Noben-Trauth, 2002; Von Stebut et al., 2003). In this line, it was demonstrated that *L. major* growth in the BALB/c is uncontrolled, leading to progressive lesions and disseminated visceral infection (Loeuillet et al., 2016; Novais et al., 2021). Accordingly, numerous studies have used BALB/c as a susceptible model for *in-vivo* infectivity and drug sensitivity assessment of cutaneous leishmaniasis (Badirzadeh et al., 2022; Fortin et al., 2014; Guimarães et al., 2021; Masic et al., 2015). We added the susceptibility of BALB/c to *L. major* with a reference in the M&M, highlighted in green.

**- Any specific reason to euthanize only 3 mice out of 9**

***Response*:** Thank you for raising this point. The minimum sample size for statistical analysis of parasite load by qPCR is three, though as the sample size increases, so does the power of the significance test. However, in some studies, this sample size was used for analyzing parasite load (Gonçalves-Oliveira et al., 2019; Jaafari et al., 2019; Nicolas et al., 2000). We conducted the quantitative real-time PCR on three randomly selected animals in each group. Moreover, the qPCR method is a validated and reliable method that is routinely used for the diagnosis of infection in patients. The references were added in the M&M to support our data (line 197, highlighted in red color).

Moreover, as stated in the M&M, before starting the treatment and at the end of the fifth week, samples from each lesion were prepared and stained with Giemsa 10% to detect the amastigotes by the light microscope. According to the previous studies, parasite loads in the skin lesion also had determined based on the WHO guidelines in all the groups using microscopy as ordinal division, including grade 0 (0 parasites in 1000 fields), grade 1+ (1–10 parasites in 1000 fields), grade 2+ (1–10 parasites in 100 fields), grade 3+ (1–10 parasites in 10 fields), grade 4+ (1–10 parasites per field), grade 5+ (10–100 parasites per field), or grade 6+ (> 100 parasites per field) (Ghosh et al., 2022; Mohtasebi et al., 2019; Pouryousef et al., 2021; WHO, 2010). The microscopy results revealed that the parasite load was significantly reduced in the groups received antileishmanial drugs compared to the untreated control group (*p*< 0.05). The group receiving EGF 4.5 µg/kg plus amphotericin B displayed the lowest amastigote number in the smears. However, since in microscopical grading, the distances among numbers of amastigotes are wide, and probability errors for their count are high, we evaluated the parasite load via qPCR on three randomly selected animals in each group. Given that the results of parasite load by qPCR agreed with the reduction of lesion size in the groups administrated EGF 4.5 µg/mL supplement, we did not include the microscopy results in the manuscript.

**Reviewer #1: The results are clearly show. Possibly too clear with the same data shown in three different ways.**

**The text to figure 5 C, D should state which group the samples came from-**

***Response:*** Thank you for the comments. We Grouped the figures 1, 2, and 3. Also, 5 C and D correspond to the control group added in the caption, (line 320, highlighted in teal color).

Reviewer #2: -Does the analysis presented match the analysis plan? Yes.

-Are the results clearly and completely presented? Yes.

-Are the figures (Tables, Images) of sufficient quality for clarity? Yes.

**Reviewer #3: - Why E1.5 and E4.5 alone groups were not created.**

**Response:** As we mentioned before, EGF is a growth factor that is used in topical or intralesional forms for the healing of chronic wounds. Several clinical trials demonstrated that EGF is not pharmacologically considered an antimicrobial agent, and its main function is to prompt wound healing (Park et al., 2017; Shin et al., 2023). Therefore, nowadays, combination therapy of EGF and antibiotics are extensively used to treat and accelerate the healing of infected wounds in clinical settings. In this context, clinical trials demonstrated that using these two reagents simultaneously, EGF significantly promotes wound healing, and silver or silver sulfadiazine can effectively prevent infection. Therefore, they jointly contribute to wound repair and shorten the healing time compared to EGF alone (Lee et al., 2021; Li et al., 2021; Zhang et al., 2021). Recently, in a self-controlled clinical trial, autologous platelet gel (APG) prepared from platelet-rich plasma (PRP) containing natural growth factors in combination with Glucantime was used in a limited number of CL patients (n=13). The combination therapy led to accelerated wound healing compared to monotherapy with Glucanime; however, the effect of APG alone was not evaluated (Shadmand et al., 2023). This was summarized and mentioned in the limitation, highlighted in yellow.

Several studies revealed that chronic refractory wounds have low levels of growth factors such as EGF; consequently, applying exogenous EGF could compensate for the diminished EGF levels and promote wound healing (Park et al., 2017). An interesting study (Montoya et al., 2018) showed that EGF expression decreased in the lesions of cutaneous leishmaniasis. As such, we aimed to test the hypothesis that the addition of EGF to the anti-*Leishmania* therapy would have a synergistic effect in the treatment of cutaneous leishmaniasis. Our results clearly showed that the simultaneous application of EGF and antileishmanial drugs improved the healing of cutaneous leishmaniasis. Interestingly, groups receiving anti-*Leishmania* drugs plus 4.5 µg/kg of EGF had lesions significantly smaller compared to the control groups receiving only Glucantime or Amphotericin B. Indeed, the antileishmanial drugs inhibit parasite growth, and exogenous EGF probably compensates for the growth factor deficiency in the CL lesion, responsible for accelerating re-[epithelialization](https://www.google.com/search?sca_esv=46b4c7e13b37a354&sca_upv=1&sxsrf=ACQVn0-xplPNk0Y1d0A4zdESs8qJ66AjDw:1712909913498&q=epithelialization&spell=1&sa=X&ved=2ahUKEwislLTXnryFAxXX9gIHHXlqBnUQkeECKAB6BAgHEAI) of the wound, and synergistically contribute to the promoting wound healing. The efficacy of EGF in healing *Leishmania* lesions was probably due to accelerating re-epithelization and reducing inflammatory responses in the wound. We argued these statements in the discussion section and also in the limitation (underlined in the text). Also, in the limitation of the study, we mentioned not including the EGF group. We suggested further analysis could be helpful regarding the effect of exogenous EGF alone on the wound healing process, the host inflammation response, and scar formation in cutaneous leishmaniasis (highlighted in yellow).

**- Why topical paromomyin group was not created which is an established treatment.**

***Response:*** Thank you for this comment.

Pentavalent antimonial is still the first-line treatment of cutaneous and visceral leishmaniasis in Iran (Firouraghi et al., 2023; Mohebali et al., 2023). On the other hand, in some countries, such as Ethiopia, the preferential first-line treatment for CL is the intramuscular administration of paromomycin (van Griensven et al., 2016). However, there were some reports of unresponsiveness of *L. tropica* to paromomycin (Shani-Adir et al., 2005). Although there were a few studies on using topical paromomycin in Iran (Asilian et al., 2003), it has not been used commonly for the treatment of CL, and Glucantime still is the first choice for the treatment of CL due to *L. major* and *L. tropica*. In addition, recently, a new formulation of topical nano liposomal amphotericin B has been recommended for the treatment with Glucantime, and in a phase 2 clinical trial, it showed a promising efficacy of 98% (Alizadeh et al., 2023; Jaafari et al., 2019; Khamesipour et al., 2022). In the present study, we aimed to discover the effect of EGF as a complementary treatment to these available drugs in accelerating the treatment of CL.

**- If 3 mice were already** **euthanized then what was the need to** **anesthetize the tail (line 213).**

***Response:*** Thank you for your comments. The mistake was corrected. The mice were euthanized the same way as the samples were prepared for qPCR because we needed the whole base of the tail for the pathological examination. The text was edited, line 217, and highlighted in pink.

**-Any specific reason to select only 3 mice per group for analysis**

***Response*:** Thank you for raising this point. The minimum sample size for statistical analysis of parasite load by qPCR is three, though as the sample size increases, so does the power of the significance test. However, in some studies, this sample size was used for analyzing parasite load (Gonçalves-Oliveira et al., 2019; Jaafari et al., 2019; Nicolas et al., 2000). We conducted the quantitative real-time PCR on three randomly selected animals in each group. Moreover, the qPCR method is a validated and reliable method that is routinely used for the diagnosis of infection in patients. The references were added to the M&M to support our data (line 197, highlighted in red).

Moreover, as stated in the M&M, before starting the treatment and at the end of the fifth week, samples from each lesion were prepared and stained with Giemsa 10% to detect the amastigotes by the light microscope. According to the previous studies, parasite loads in the skin lesion also had determined based on the WHO guidelines in all the groups using microscopy as ordinal division, including grade 0 (0 parasites in 1000 fields), grade 1+ (1–10 parasites in 1000 fields), grade 2+ (1–10 parasites in 100 fields), grade 3+ (1–10 parasites in 10 fields), grade 4+ (1–10 parasites per field), grade 5+ (10–100 parasites per field), or grade 6+ (> 100 parasites per field) (Ghosh et al., 2022; Mohtasebi et al., 2019; Pouryousef et al., 2021; WHO, 2010). The microscopy results revealed that the parasite load was significantly reduced in the groups that received antileishmanial drugs compared to the untreated control group (*p*< 0.05). The group receiving EGF 4.5 µg/kg plus amphotericin B displayed the lowest amastigote number in the smears. However, since in microscopical grading, the distances among numbers of amastigotes are wide, and probability errors for their count are high, we evaluated the parasite load via qPCR on three randomly selected animals in each group. Given that the results of parasite load by qPCR agreed with the reduction of lesion size in the groups administrated EGF 4.5 µg/mL supplement, we did not include the microscopy results in the manuscript.

**- Why intralesional meglumine antimoniate group was not created**

***Response:*** Since the EGF was injected by subcutaneous (CS) route every other day for five weeks, we used intramuscular (IM) instead of an intralesional route for injection of Glucantime to avoid the extra manipulation of the lesion and also avoid probable side effects such as necrosis, localized pruritus which could affect the results (Brito et al., 2019; Esfandiarpour et al., 2012). Also, in several studies, intramuscular injection of Glucantime was used in animal models (Henao et al., 2004; Mohtasebi et al., 2019; Tiuman et al., 2012).

**- Why lesions were not observed up to 6 months (24 weeks) when almost half of the lesions in L. major show some self-healing.**

***Response:*** Thank you for raising this point. As stated before, L. major is usually self-healing in humans, and after 4-6 months, it is recovered. However, several strains such as BALB/c, SWR/J, and P/J are susceptible to different *Leishmania* species and develop severe non-healing systemic disease affecting the skin and visceral organs (Kurey et al., 2009; Sacks and Noben-Trauth, 2002; Von Stebut et al., 2003). In this line, it was demonstrated that *L. major* growth in the BALB/c is uncontrolled, leading to progressive lesions and disseminated visceral infection (Loeuillet et al., 2016; Novais et al., 2021). Accordingly, numerous studies have used BALB/c as a susceptible model for in-vivo infectivity and drug sensitivity assessment of cutaneous leishmaniasis (Badirzadeh et al., 2022; Fortin et al., 2014; Guimarães et al., 2021; Masic et al., 2015). We added the susceptibility of BALB/c to *L. major* with a reference in the M&M, highlighted in green.

In the present study, we used the BALB/c as a suspectable animal model for CL caused by L. major to investigate the effect of EGF combined with anti-leishmanial drugs to prevent the progression of lesions and accelerate the CL treatment. As shown in untreated control and Normal Saline control groups, the lesion sizes remarkably increased without chemotherapy, and there was no self-healing. On the other hand, groups that received anti-leishmanial drugs exhibited prevention from the lesion and a gradual decrease in lesion size compared to control groups. It should be mentioned that in several studies, antimonial compounds used between 20 to 35 days (Mohtasebi et al., 2019; Ramos et al., 2020) and topical Amphotericin administrated for 28-30 days (Jaafari et al., 2019; Mousavi et al., 2022). Consequently, according to other studies, after finishing the treatment course and as the significant reduction was demonstrated in the lesion size of test groups from the fourth week, to avoid losing the mice in the control groups due to the uncontrolled progressive lesion (up to 20 mm^2^), we finished the experiment at the end of the fifth week.

**Reviewer #1:** **The conclusion would as stated above benefit from the inclusion of an EFG treated group. It would also be high relevant to address the dissemination of L. major, know to occur in Balb/c mice.**

***Response:*** Thank you for raising this point. In the discussion, we stated the progression and dissemination of *L. major* occurred in susceptible BALB/C. Also, we mentioned that the administration of EFG with the antileishmanial drugs could prevent lesion progression and accelerate lesion treatment, which was written in purple.

**Importantly the effect on parasite load need more data (n=3, as indicated is not sufficient for conclusion) and it would also be relevant to know that the parasites are dead and not incapsulated in a closing wound.**

***Response*:** Thank you for raising this point. The minimum sample size for statistical analysis of parasite load by qPCR is three, though as the sample size increases, so does the power of the significance test. However, in some studies, this sample size was used for analyzing parasite load (Gonçalves-Oliveira et al., 2019; Jaafari et al., 2019; Nicolas et al., 2000). We conducted the quantitative real-time PCR on three randomly selected animals in each group. Moreover, the qPCR method is a validated and reliable method that is routinely used for the diagnosis of infection in patients. The references were added in the M&M to support our data (line 197, highlighted in red color).

Moreover, as stated in the M&M, before starting the treatment and at the end of the fifth week, samples from each lesion were prepared and stained with Giemsa 10% to detect the amastigotes by the light microscope. According to the previous studies, parasite loads in the skin lesion also had determined based on the WHO guidelines in all the groups using microscopy as ordinal division, including grade 0 (0 parasites in 1000 fields), grade 1+ (1–10 parasites in 1000 fields), grade 2+ (1–10 parasites in 100 fields), grade 3+ (1–10 parasites in 10 fields), grade 4+ (1–10 parasites per field), grade 5+ (10–100 parasites per field), or grade 6+ (> 100 parasites per field) (Ghosh et al., 2022; Mohtasebi et al., 2019; Pouryousef et al., 2021; WHO, 2010). The microscopy results revealed that the parasite load was significantly reduced in the groups received antileishmanial drugs compared to the untreated control group (*p*< 0.05). The group receiving EGF 4.5 µg/kg plus amphotericin B displayed the lowest amastigote number in the smears. However, since in microscopical grading, the distances among numbers of amastigotes are wide, and probability errors for their count are high, we evaluated the parasite load via qPCR on three randomly selected animals in each group. Given that the results of parasite load by qPCR agreed with the reduction of lesion size in the groups administrated EGF 4.5 µg/mL supplement, we did not include the microscopy results in the manuscript.

Regarding the comments identifying that the parasites were dead and not encapsulated in a closing wound, as we determined in the qPCR and microscopy techniques, the parasite load remarkably reduced in the groups administrated antileishmanial drug plus EGF 4.5 µg/kg. As we extracted most the lesions (20-25 mg) for DNA extraction, the exact number of the parasite in the lesion was determined. Although a slight parasite load was still identified in groups administrated antileishmanial drug plus EGF 4.5 µg/kg, repairing tissue with the absence of inflammatory cells and amastigote was detected in the pathologic sections prepared from the edge of the lesion where the wound was repaired. Moreover, as shown in Fig 3, lesion appearance in the groups that received antileishmanial plus EGF 4.5 µg/kg was smaller, and epithelialization was obviously detected at the edge of the lesion compared to monotherapy with Glucantime or amphotericin B. These findings were especially predominant in the group that received amphotericin B plus EGF 4.5 µg/kg. In consensus, our results indicated that the EGF 4.5 µg/kg could prompt wound healing in CL. This statement was added to the discussion and highlighted in turquoise.

**Limitations of the study could be discussed more.**

***Response:*** Thank you for this comment; the limitation was added at the end of the discussion, highlighted in yellow.

Reviewer #2: -Are the conclusions supported by the data presented? Yes.

-Are the limitations of analysis clearly described? Yes.

-Do the authors discuss how these data can be helpful to advance our understanding of the topic under study? Yes.

-Is public health relevance addressed? Yes.

**Reviewer #3: - Line 51- A correction is needed. Amphotericin B or its lipid complexes are not the pillar choice for treatment of CL treatment.**

***Response:*** The text was edited (line 51).

**- Generally, if the lesion is less than 4 cm, L. major lesions are self-curing in 4-6 months. In this study, none of the lesions showed any signs of self-cure. On the contrary lesions have increased in size in C and N groups.**

***Response:* *Response:*** Thank you so much for your question.

Genetic determinants regulate susceptibility and resistance to Leishmania infection in humans and mice. Usually, *L. major* is self-healing in humans and recovered after 4-6 months. However, several strains, such as BALB/c, SWR/J, and P/J, are susceptible to different *Leishmania* species and develop severe non-healing systemic diseases affecting skin and visceral organs (Kurey et al., 2009; Sacks and Noben-Trauth, 2002). In this line, it was demonstrated that *L. major* growth in the BALB/c is uncontrolled/unrestrained, leading to progressive lesions and disseminated visceral infection (Loeuillet et al., 2016; Novais et al., 2021). Accordingly, numerous studies have used BALB/c as a susceptible model for in-vivo infectivity and drug sensitivity assessment of cutaneous leishmaniasis (Badirzadeh et al., 2022; Fortin et al., 2014; Guimarães et al., 2021; Masic et al., 2015). We added a reference for the susceptibility of BALB/c mice to *L. major*, highlighted in green.

In the present study, we used the BALB/c as a suspectable animal model for CL caused by *L. major* to investigate the effect of EGF combined with the anti-leishmanial drug in preventing the progression of lesion and accelerating the CL treatment. As shown in the no-treatment control and Normal Saline control groups, without chemotherapy, the lesion sizes and parasite load were remarkably increased with no self-healing. On the other hand, groups that received anti-leishmanial drugs exhibited a gradual decrease in lesion size and parasite load compared to control groups.

**- It is not explained why lesions in G have increased in size despite of receiving the full course of meglumine antimoniate which is an established and very effective CL treatment.**

***Response:*** Thank you for this comment. In several studies in BALB/c, which is susceptible to *Leishmania* species, anti-leishmanial drugs such as Glucantime and amphotericin B limit the lesion progression and prevent the development of lesions, leading to smaller lesion size compared to untreated control groups after 4 or 5-week treatment (Badirzadeh et al., 2022; Gonçalves-Oliveira et al., 2019; Mohtasebi et al., 2019; Ramos et al., 2020; Sombra Santos et al., 2023).

In the present study, the trend of lesion size after starting the chemotherapy was slightly progressive in the first weeks as compared to the initial lesion size at the starting point due to the time course of antileishmanial response; however, after the second week, the lesion sizes were significantly smaller in all the groups received antileishmanial drugs compared to untreated groups (C, N). Antileishmanial chemotherapy significantly prevented the progression of lesions after the third week compared with untreated control, although the development of lesion size was slightly increasing in all the groups during the first weeks. This trend of drug response was also detected in lesion size during chemotherapy within 4-5 weeks in previous studies (Badirzadeh et al., 2022; Gonçalves-Oliveira et al., 2019; Mohtasebi et al., 2019; Ramos et al., 2020; Sombra Santos et al., 2023). This brief explanation of the trend of chemotherapy added to the discussion, highlighted in gray color.

In some studies, antimonial therapy continued for up to 50 days or higher doze of antimonial (100 mg/mL) was used to complete wound healing; however, we did not use this scheme due to the possibility of the drug toxicity and thereby losing the animals (Jabini et al., 2015; Ramos et al., 2020; Sombra Santos et al., 2023). Here, we used the standard 21-day course of treatment to compare the EGF supplement.

**- Except S+E4.5 and G+E4.5 groups, the other 6 groups have increased in size of lesions week by week, and no explanation is given. It seems the other 6 groups were refractory to the treatment which is difficult to understand.**

***Response:*** As mentioned earlier, the trend of lesion development after chemotherapy was progressive in the first weeks as compared to the initial lesion size at the starting point; however, after the second week, the lesion sizes in all the groups receiving antileishmanial drugs were significantly smaller compared to untreated groups (C, N) (Table 2). In harmony with previous studies, antileishmanial chemotherapy significantly prevented the progression of lesions after the third week in comparison with untreated control, although lesion size was increased compared to the initial lesion size at first week (Badirzadeh et al., 2022; Gonçalves-Oliveira et al., 2019; Mohtasebi et al., 2019; Ramos et al., 2020; Sombra Santos et al., 2023). This brief explanation of the trend of chemotherapy added to the result and discussion, highlighted in gray color.

Moreover, in the present study, EGF 1.5 µg/kg was ineffective, and its results were the same as monotherapy with Glucantime or Amphotericin B. On the other hand, administration of EGF 4.5 µg/kg plus antileishmanial drug after the fourth week significantly reduced mean lesion size compared to monotherapy with antileishmanial drugs. The possible reason could be the time course of EGF response, which is marked by the re-epithelialization of the wound. Several studies demonstrated that after injection of EGF every other day in chronic wounds, most patients showed wound healing only after 4-5 weeks of treatment, i.e., after 10-12 injections (Fernández-Montequín et al., 2009). The same phenomenon was observed here, as injection of EGF 4.5 µg/kg plus antileishmanial drug showed the maximum effect in reducing the lesion size after four weeks of treatment. This statement was added to the result and discussion sections and written in red.

**- Can decrease in size of the lesions in S+E4.5 and G+E4.5 groups be attributed to self-healing? Also had lesions been observed beyond 5 weeks in the remaining 6 groups, lesions might have shown decrease in size?**

***Response:*** Thank you so much for your question.

As mentioned before, BALB/c is susceptible to *L. major,* leading to the development of progressive severe non-healing lesions and disseminated visceral infection (Kurey et al., 2009; Loeuillet et al., 2016; Novais et al., 2021; Sacks and Noben-Trauth, 2002; Von Stebut et al., 2003). Accordingly, numerous studies have used BALB/c as a susceptible model for in-vivo infectivity and drug sensitivity assessment of cutaneous leishmaniasis (Badirzadeh et al., 2022; Fortin et al., 2014; Guimarães et al., 2021; Masic et al., 2015). In the present study, we used the BALB/c as a suspectable animal model for CL caused by L. major to investigate the effect of EGF combined with anti-leishmanial drug to prevent the progression of lesions and accelerate the CL treatment. As shown in Fig1, in untreated and Normal Saline control groups, without chemotherapy, the lesion sizes and parasite load were remarkably increased with no self-healing. On the other hand, groups that received anti-leishmanial drug exhibited gradual inhibition of lesion development, leading to a reduction in lesion size and parasite load compared to the untreated groups. Administration of EGF 4.5 µg/kg plus antileishmanial drug after the fourth week led to a significant reduction in mean lesion size compared to monotherapy with antileishmanial drugs.

It should be mentioned that in several studies, antimonial compounds used between 20 to 35 days in animal models (Mohtasebi et al., 2019; Ramos et al., 2020) and topical Amphotericin administrated for 28-30 days (Jaafari et al., 2019; Mousavi et al., 2022) so that in these surveys the treatment course limited to 4-6 weeks.

Consequently, according to previous studies, after finishing the treatment course and as the significant reduction was demonstrated in the lesion size of test groups from the fourth week, to avoid losing the mice in the control groups due to the uncontrolled progressive lesion (up to 20 mm^2^), we finished the experiment at the end of the fifth week.

Although in the 5-week scheme, reduction in the lesion size and parasite load was attained, extending the treatment course would be interesting to see if complete wound healing is obtained in groups receiving EGF. We stated in the limitation of the study that in further analysis, the treatment course can be extended to gain complete healing, highlighted in yellow.

**- It is not clear why lesions increased in size up to the fourth week in G+4.5 and then suddenly decreased in the fifth week. On the contrary in G+E1.5 lesions have continued to increase in size throughout the five weeks' period.**

***Response:*** In the present study using EGF 1.5 µg/kg was ineffective, and the results were the same as monotherapy with Glucantime or Amphotericin B, which significantly prevented the progression of lesions after the fourth week in comparison with untreated control according to the previous reports (Badirzadeh et al., 2022; Gonçalves-Oliveira et al., 2019; Mohtasebi et al., 2019; Ramos et al., 2020; Sombra Santos et al., 2023).

On the other hand, administration of EGF 4.5 µg/kg plus antileishmanial drug after the fourth week significantly reduced mean lesion size compared to monotherapy with antileishmanial drugs. The possible reason could be due to the time course of EGF response, as several studies demonstrated that EGF gradually, after the fourth week of injection, induces reepithelization and accelerate wound healing (Fernández-Montequín et al., 2009; Park et al., 2018; Rahim et al., 2022).

In this line, several clinical trial studies demonstrated that after intralesional injection of a commercial epidermal growth factor‐based formulation (Heberprot‐P) every other day in chronic and non-healing diabetic foot ulcers, most patients showed ulcer size reduction and signs of wound healing only after 4-5 weeks of treatment, i.e., after 10-12 injection. (Camacho-Rodríguez et al., 2018; Fernández-Montequín et al., 2009). The same phenomenon was observed here, as injection of EGF 4.5 µg/kg plus antileishmanial drug showed the maximum effect in reducing the lesions after four weeks of treatment. This statement was added to the result and discussion sections and written in red.

Notably, the optimum intralesional dose of EGF for healing diabetic foot ulcer is 1 µg/kg (Camacho-Rodríguez et al., 2018; Ryu et al., 2009). We used two doses of 1.5 and 4.5 µg/kg to compensate for faster drug metabolism in rodents and lower efficacy of human EGF in mice. Our results clearly showed that 1.5 µg/kg of EGF was insufficient for wound healing in mice, but the 4.5 µg/kg dose reduced the lesion sizes in combination with antileishmanial drugs. We added this statement in the discussion, written in red color

**- Difficult to interpret the results from table**

***Response:*** The table description was edited in the text and written in blue.

--------------------

<b>Editorial and Data Presentation Modifications?</b></br><br/>

Use this section for editorial suggestions as well as relatively minor modifications of existing data that would enhance clarity. If the only modifications needed are minor and/or editorial, you may wish to recommend "Minor Revision" or "Accept".

**Reviewer #1: The figures 1-3 show the same thing and should be combined into one figure (1a-c). Also the figure 1 shows the same as table 2 and is repetitive. The table is the more informative.**

***Response:*** Thanks for the relevant comment. The figures were combined. As we wanted to show the trend of chemotherapy in Fig 1, if the reviewer accepted it, we combined the figures.

**Reviewer #2: Apart from a few old references that need to be updated and some English terms that need to be improved, the article is well-written and reliably addresses the results discussed.**

***Response:*** Thank you for your comments. We went through the entire manuscript to edit and update the references.

Reviewer #3: (No Response)

--------------------

<b>Summary and General Comments</b></br></br>

Use this section to provide overall comments, discuss strengths/weaknesses of the study, novelty, significance, general execution and scholarship. You may also include additional comments for the author, including concerns about dual publication, research ethics, or publication ethics. If requesting major revision, please articulate the new experiments that are needed.

**Reviewer #1: Therapies aiming at reducing the scaring that can be caused by Leishmania are desirable for many and the scope of this study is relevant.**

**As is stands the scope of the study is very narrow, with one large experiment performed and with the amount of data presented being limited. More extensive analysis of parasite load,** **dissemination, tissue responses etc would increase the value of the study. Importantly understandning how EFG alone affects parasite load, dissemination and lesion size is highly relevant.**

***Response:*** Thank you for your insightful comment.

For the first time, we report that administration of EGF 4.5 µg/kg combined with antileishmanial drugs displayed superior healing efficacy in the treatment of CL, compared to antileishmanial drugs alone or combined with EGF 1.5 µg/kg. Lesion healing was evaluated by measuring lesion size, microscopic examination of the lesions for parasites, and quantifying parasite load using qPCR and pathology. As EGF is reported to exert an anti-inflammatory response, we suggest quantifying pro- and anti-inflammatory cytokines in the lesions to understand the effect of EGF on lesion healing. We did not include a control EGF group because treatment of CL requires effective antileishmanial therapy; however, we suggest the addition of a control EGF group in future studies. The fact that administration of EGF 4.5 µg/kg with two different antileishmanial drugs effectively reduced the lesion size and parasite load in CL warrants further investigating EGF administration, e.g., topical and intralesional, in treating CL, and the possible mechanisms for such an effect. Consequently, it can be suggested as a supplement to accelerate CL treatment. Nevertheless, further analysis and future clinical trials are needed to confirm our data. The limitation was added to the discussion and highlighted in yellow.

Reviewer #2: Not applicable.

**Reviewer #3: Major revisions and explanations needed**

--------------------

PLOS authors have the option to publish the peer review history of their article (<a href="https://journals.plos.org/plosntds/s/editorial-and-peer-review-process#loc-peer-review-history" target="_blank">what does this mean?</a>). If published, this will include your full peer review and any attached files.<br><br>

If you choose "no", your identity will remain anonymous but your review may still be made public.<br><br>

<b>Do you want your identity to be public for this peer review?</b> For information about this choice, including consent withdrawal, please see our <a href="https://www.plos.org/privacy-policy" target="_blank">Privacy Policy</a>.

Reviewer #1: No

Reviewer #2: No

Reviewer #3: Yes: Saurabh Jain

**References**

Alizadeh, Z., Shirzadi, M.R., Hassanpour, G.R., Keshavarz, H., Mohebali, F., Eskandari, S.E., Zeinali, M., Shirmohammad, S., Mohebali, M., 2023. Field Efficacy of Topical Nano-Liposomal Amphotericin B (Sina Ampholeish®) Alone or in Combination with Glucantime® and Cryotherapy on Human Cutaneous Leishmaniasis. Iranian journal of parasitology 18, 419-426.

Asilian, A., Jalayer, T., Nilforooshzadeh, M., Ghassemi, R.L., Peto, R., Wayling, S., Olliaro, P., Modabber, F., 2003. Treatment of cutaneous leishmaniasis with aminosidine (paromomycin) ointment: double-blind, randomized trial in the Islamic Republic of Iran. Bull World Health Organ 81, 353-359.

Badirzadeh, A., Najm, M., Hemphill, A., Alipour, M., Hasanpour, H., Masoori, L., Karimi, P., 2022. Effects of negative air ions (NAIs) on Leishmania major: A novel tool for treatment of zoonotic cutaneous leishmaniasis (ZCL). PLoS One 17, e0274124.

Blackwell, J.M., Fakiola, M., Ibrahim, M.E., Jamieson, S.E., Jeronimo, S.B., Miller, E.N., Mishra, A., Mohamed, H.S., Peacock, C.S., Raju, M., Sundar, S., Wilson, M.E., 2009. Genetics and visceral leishmaniasis: of mice and man. Parasite Immunol 31, 254-266.

Brito, N.C., Machado de Assis, T.S., Rabello, A., Cota, G., 2019. Intralesional infiltration versus parenteral use of meglumine antimoniate for treatment of cutaneous leishmaniasis: A cost-effectiveness analysis. PLoS Negl Trop Dis 13, e0007856.

Camacho-Rodríguez, H., Guillen-Pérez, I.A., Roca-Campaña, J., Baldomero-Hernández, J.E., Tuero-Iglesias Á, D., Galván-Cabrera, J.A., Rodriguez-Cordero, M., Palenzuela-Gardón, D.O., Berlanga-Acosta, J., Novoa-Pérez, L.I., 2018. Heberprot-P's Effect on Gene Expression in Healing Diabetic Foot Ulcers. MEDICC review 20, 10-14.

Esfandiarpour, I., Farajzadeh, S., Rahnama, Z., Fathabadi, E.A., Heshmatkhah, A., 2012. Adverse effects of intralesional meglumine antimoniate and its influence on clinical laboratory parameters in the treatment of cutaneous leishmaniasis. Int J Dermatol 51, 1221-1225.

Fernández-Montequín, J.I., Betancourt, B.Y., Leyva-Gonzalez, G., Mola, E.L., Galán-Naranjo, K., Ramírez-Navas, M., Bermúdez-Rojas, S., Rosales, F., García-Iglesias, E., Berlanga-Acosta, J., Silva-Rodriguez, R., Garcia-Siverio, M., Martinez, L.H., 2009. Intralesional administration of epidermal growth factor-based formulation (Heberprot-P) in chronic diabetic foot ulcer: treatment up to complete wound closure. Int Wound J 6, 67-72.

Firouraghi, N., Bergquist, R., Fatima, M., Mohammadi, A., Hamer, D.H., Shirzadi, M.R., Kiani, B., 2023. High-risk spatiotemporal patterns of cutaneous leishmaniasis: a nationwide study in Iran from 2011 to 2020. Infectious diseases of poverty 12, 49.

Fortin, A., Caridha, D.P., Leed, S., Ngundam, F., Sena, J., Bosschaerts, T., Parriott, S., Hickman, M.R., Hudson, T.H., Grogl, M., 2014. Direct comparison of the efficacy and safety of oral treatments with oleylphosphocholine (OlPC) and miltefosine in a mouse model of L. major cutaneous leishmaniasis. PLoS Negl Trop Dis 8, e3144.

Ghosh, P., Chowdhury, R., Maruf, S., Picado, A., Hossain, F., Owen, S.I., Nath, R., Baker, J., Hasnain, M.G., Shomik, M.S., Ghosh, D., Rashid, M., Rashid, M.U., Sagar, S.K., Rahat, M.A., Basher, A., Nath, P., Edwards, T., Andrews, J.R., Duthie, M.S., de Souza, D.K., Adams, E.R., Ndungu, J., Cruz, I., Mondal, D., 2022. Gauging the skin resident Leishmania parasites through a loop mediated isothermal amplification (LAMP) assay in post-kala-azar dermal leishmaniasis. Scientific reports 12, 18069.

Gonçalves-Oliveira, L.F., Souza-Silva, F., de Castro Côrtes, L.M., Veloso, L.B., Santini Pereira, B.A., Cysne-Finkelstein, L., Lechuga, G.C., Bourguignon, S.C., Almeida-Souza, F., da Silva Calabrese, K., Ferreira, V.F., Alves, C.R., 2019. The combination therapy of meglumine antimoniate and oxiranes (epoxy-α-lapachone and epoxymethyl-lawsone) enhance the leishmanicidal effect in mice infected by Leishmania (Leishmania) amazonensis. International journal for parasitology. Drugs and drug resistance 10, 101-108.

Guimarães, T.T., Gomes, S.M.R., Albuquerque, R., Lima, A.K.C., Braga, G.F., Souza, J.B., Assis, M., Brito, A.C.S., Santos, R.F., Da Silva, T., Siqueira, L.M., Ventura, B.D., Rodrigues, L.S., Terra, R., Da Silva, S.A.G., Dutra, P.M.L., 2021. Chronic Aerobic Training at Different Volumes in the Modulation of Macrophage Function and in vivo Infection of BALB/c Mice by Leishmania major. Front Microbiol 12, 734355.

Henao, H.H., Osorio, Y., Saravia, N.G., Gómez, A., Travi, B., 2004. [Efficacy and toxicity of pentavalent antimonials (Glucantime and Pentostam) in an American cutaneous leishmaniasis animal model: luminometry application]. Biomedica 24, 393-402.

Jaafari, M.R., Hatamipour, M., Alavizadeh, S.H., Abbasi, A., Saberi, Z., Rafati, S., Taslimi, Y., Mohammadi, A.M., Khamesipour, A., 2019. Development of a topical liposomal formulation of Amphotericin B for the treatment of cutaneous leishmaniasis. International journal for parasitology. Drugs and drug resistance 11, 156-165.

Jabini, R., Jaafari, M.R., Vahdati Hasani, F., Ghazizadeh, F., Khamesipour, A., Karimi, G., 2015. Effects of combined therapy with silymarin and glucantime on leishmaniasis induced by Leishmania major in BALB/c mice. Drug Res (Stuttg) 65, 119-124.

Khamesipour, A., Mohammadi, A., Jaafari, M., Eskandari, S., Tasbihi, M., Javadi, A., Afshari, F., Mortazavi, H., Firooz, A., 2022. Pilot study of safety and efficacy of topical liposomal amphotericin B for cutaneous leishmaniasis caused by Leishmania major in Islamic Republic of Iran. Eastern Mediterranean health journal = La revue de sante de la Mediterranee orientale = al-Majallah al-sihhiyah li-sharq al-mutawassit 28, 658-663.

Kurey, I., Kobets, T., Havelková, H., Slapnicková, M., Quan, L., Trtková, K., Grekov, I., Svobodová, M., Stassen, A.P., Hutson, A., Demant, P., Lipoldová, M., 2009. Distinct genetic control of parasite elimination, dissemination, and disease after Leishmania major infection. Immunogenetics 61, 619-633.

Lee, Y.H., Hong, Y.L., Wu, T.L., 2021. Novel silver and nanoparticle-encapsulated growth factor co-loaded chitosan composite hydrogel with sustained antimicrobility and promoted biological properties for diabetic wound healing. Materials science & engineering. C, Materials for biological applications 118, 111385.

Li, S., Liu, Y., Huang, Z., Kou, Y., Hu, A., 2021. Efficacy and safety of nano-silver dressings combined with recombinant human epidermal growth factor for deep second-degree burns: A meta-analysis. Burns : journal of the International Society for Burn Injuries 47, 643-653.

Loeuillet, C., Bañuls, A.L., Hide, M., 2016. Study of Leishmania pathogenesis in mice: experimental considerations. Parasit Vectors 9, 144.

Masic, A., Valencia Hernandez, A.M., Hazra, S., Glaser, J., Holzgrabe, U., Hazra, B., Schurigt, U., 2015. Cinnamic Acid Bornyl Ester Derivatives from Valeriana wallichii Exhibit Antileishmanial In Vivo Activity in Leishmania major-Infected BALB/c Mice. PLoS One 10, e0142386.

Mohebali, M., Edrissian, G., Akhoundi, B., Shirzadi, M., Hassanpour, G., Behkar, A., Rassi, Y., Hajjaran, H., Keshavarz, H., Gouya, M.M., Arshi, S., Zeinali, M., Zarei, Z., Sharifi, I., Kakooei, Z., 2023. Visceral Leishmaniasis in Iran: An Update on Epidemiological Features from 2013 to 2022. Iranian journal of parasitology 18, 279-293.

Mohtasebi, S., Mohebali, M., Elikaee, S., Akhoundi, B., Foroushani, A.R., Teimouri, A., Yarizadeh, H., 2019. In vitro and in vivo anti-parasitic activity of biogenic antimony sulfide nanoparticles on Leishmania major (MRHO/IR/75/ER). Parasitology research 118, 2669-2678.

Montoya, A., Yepes, L., Bedoya, A., Henao, R., Delgado, G., Vélez, I.D., Robledo, S.M., 2018. Transforming Growth Factor Beta (TGFβ1) and Epidermal Growth Factor (EGF) as Biomarkers of Leishmania (V) braziliensis Infection and Early Therapeutic Response in Cutaneous Leishmaniasis: Studies in Hamsters. Front Cell Infect Microbiol 8, 350.

Mousavi, P., Rahimi Esboei, B., Pourhajibagher, M., Fakhar, M., Shahmoradi, Z., Hejazi, S.H., Hassannia, H., Nasrollahi Omran, A., Hasanpour, H., 2022. Anti-leishmanial effects of resveratrol and resveratrol nanoemulsion on Leishmania major. BMC microbiology 22, 56.

Nicolas, L., Sidjanski, S., Colle, J.H., Milon, G., 2000. Leishmania major reaches distant cutaneous sites where it persists transiently while persisting durably in the primary dermal site and its draining lymph node: a study with laboratory mice. Infection and immunity 68, 6561-6566.

Novais, F.O., Amorim, C.F., Scott, P., 2021. Host-Directed Therapies for Cutaneous Leishmaniasis. Front Immunol 12, 660183.

Park, J.W., Hwang, S.R., Yoon, I.S., 2017. Advanced Growth Factor Delivery Systems in Wound Management and Skin Regeneration. Molecules (Basel, Switzerland) 22.

Park, K.H., Han, S.H., Hong, J.P., Han, S.K., Lee, D.H., Kim, B.S., Ahn, J.H., Lee, J.W., 2018. Topical epidermal growth factor spray for the treatment of chronic diabetic foot ulcers: A phase III multicenter, double-blind, randomized, placebo-controlled trial. Diabetes research and clinical practice 142, 335-344.

Pouryousef, A., Eslami, E., Shahriarirad, S., Zoghi, S., Emami, M., Cheraghi, M.R., Zamiri, B., Mohammadi-Samanii, S., Sarkari, B., 2021. Effects of topical gel formulation of Ficus carica latex on cutaneous leishmaniasis induced by Leishmania major in BALB/c mice. BMC research notes 14, 199.

Rahim, F., Yan, X., Shah, J.A., Bibi, N., Khan, Z.U., Nawaz, S., Ming, Y., 2022. Epidermal growth factor outperforms placebo in the treatment of diabetic foot ulcer: a meta-analysis. F1000Research 11, 773.

Ramos, T.D., Silva, J.D., da Fonseca-Martins, A.M., da Silveira Pratti, J.E., Firmino-Cruz, L., Maciel-Oliveira, D., Dos-Santos, J.S., Tenorio, J.I.N., de Araujo, A.F., Freire-de-Lima, C.G., Diaz, B.L., Cruz, F.F., Rocco, P.R.M., de Matos Guedes, H.L., 2020. Combined therapy with adipose tissue-derived mesenchymal stromal cells and meglumine antimoniate controls lesion development and parasite load in murine cutaneous leishmaniasis caused by Leishmania amazonensis. Stem cell research & therapy 11, 374.

Ryu, S.H., Moon, S.Y., Yang, Y.J., Moon, S.R., Hong, J.P., Choi, J., Lee, S.W., 2009. Recombinant human epidermal growth factor accelerates the proliferation of irradiated human fibroblasts and keratinocytes in vitro and in vivo. Journal of radiation research 50, 545-552.

Sacks, D., Noben-Trauth, N., 2002. The immunology of susceptibility and resistance to Leishmania major in mice. Nature reviews. Immunology 2, 845-858.

Shadmand, E., Solhjoo, K., Taghipour, A., Tayer, A.H., Sadeghi, F., Meshkin, A., 2023. Healing effects of autologous platelet gel and growth factors on cutaneous leishmaniasis wounds in addition to antimony; a self-controlled clinical trial with randomized lesion assignment. BMC research notes 16, 200.

Shani-Adir, A., Kamil, S., Rozenman, D., Schwartz, E., Ramon, M., Zalman, L., Nasereddin, A., Jaffe, C.L., Ephros, M., 2005. Leishmania tropica in northern Israel: a clinical overview of an emerging focus. J Am Acad Dermatol 53, 810-815.

Shin, S.H., Koh, Y.G., Lee, W.G., Seok, J., Park, K.Y., 2023. The use of epidermal growth factor in dermatological practice. Int Wound J 20, 2414-2423.

Sombra Santos, A., de Castro Rodrigues, N.L., Marciano Fonseca, F.R., Braga Fayão Oliveira, N., Oliveira Loucard, B., Fernandes Heredia, F., Albuquerque Gomes Nogueira, T.N., Monteiro Abreu, T., Silva Dos Santos, H., Holanda Teixeira, E., Almeida Moreira Leal, L.K., Brandim Gomes, R.B., Romero Teixeira, C., Teixeira, M.J., 2023. Leishmanicidal and healing effects of 3β,6β,16β-trihydroxy lup-20 (29)-ene isolated from Combretum leprosum on Leishmania braziliensis infection in vitro and in vivo. PloS one 18, e0287665.

Tiuman, T.S., Brenzan, M.A., Ueda-Nakamura, T., Filho, B.P., Cortez, D.A., Nakamura, C.V., 2012. Intramuscular and topical treatment of cutaneous leishmaniasis lesions in mice infected with Leishmania amazonensis using coumarin (-) mammea A/BB. Phytomedicine 19, 1196-1199.

van Griensven, J., Gadisa, E., Aseffa, A., Hailu, A., Beshah, A.M., Diro, E., 2016. Treatment of Cutaneous Leishmaniasis Caused by Leishmania aethiopica: A Systematic Review. PLoS Negl Trop Dis 10, e0004495.

Von Stebut, E., Ehrchen, J.M., Belkaid, Y., Kostka, S.L., Molle, K., Knop, J., Sunderkotter, C., Udey, M.C., 2003. Interleukin 1alpha promotes Th1 differentiation and inhibits disease progression in Leishmania major-susceptible BALB/c mice. J Exp Med 198, 191-199.

WHO, 2010. Control of the leishmaniases: report of a meeting of the WHO Expert Commitee on the Control of Leishmaniases, Geneva, 22-26 March 2010. World Health Organization Technical Report Series; no. 949 <https://iris.who.int/handle/10665/44412>.

Zhang, K., Li, Y., He, J., Xu, J., Wan, Y., Wan, S., Wang, R., Zeng, Q., 2021. Therapeutic Effect of Epidermal Growth Factor Combined With Nano Silver Dressing on Diabetic Foot Patients. Frontiers in pharmacology 12, 627098.
