# Supplementary material for: Contribution of epidermal growth factor (EGF) in the treatment of cutaneous leishmaniasis caused by Leishmania major in BALB/c mice
Source: PLoS Negl Trop Dis. 2025 Jan 14;19(1):e0012765. doi: 10.1371/journal.pntd.0012765 (PMC11771879; doi:10.1371/journal.pntd.0012765)
Supplement: Attachment — Submitted filename: Respond to reviewer - second one -12 september 2024.docx [file pntd.0012765.s005.docx]

**Dear Prof. Abhay R Satoskar**

**Editor of**

**PLOS Neglected Tropical Diseases	12-September-2024**

Regarding your email concerning our manuscript entitled: "**Contribution of epidermal growth factor (EGF) in the treatment of cutaneous leishmaniasis caused by *Leishmania major* in BALB/c mice**" I would like to thank you for your informative email and the reviewers for their valuable and critical comments. I would like to inform you that the manuscript has been entirely edited, and all the following corrections and changes were made based on reviewers' suggestions. New and increased subjects were highlighted in the manuscript.

We hope the changes listed below meet the publication requirements.

I sincerely appreciate your consideration and look forward to hearing from you.

Sincerely Yours,

Mehdi Mohebali (DVM, MPH, Ph.D),

Corresponding author, Professor of Medical Parasitology

Department of Parasitology & Mycology, Tehran University of Medical Sciences,

Tel/Fax: +9821-88951392

**Reviewer #3: 1. It is still not clear in the manuscript that why the lesions in the Glucantime treated mice continued to increase in spite of chemotherapy.**

**Response:** Thank you for your valuable comment. We investigated the chemotherapy in BALB/c mice, which are highly susceptible to *Leishmania major* due to the early secretion of IL-4 and Th2-type response leading to nonhealing progressive lesions (Nishikomori, Gurunathan et al. 2001).

Here, we used Glucantime at a dosage of 20 mg sb^5^/kg for 21 days (IM injection) based on the standard protocol for treating human CL due to *L. major* in Iran. In this line, several researchers have used this dosage as a standard in the BALB/c model (Jabini, Jaafari et al. 2015, Moosavian, Fallah et al. 2019, Mostafavi, Sharifi et al. 2019, Rahimi, Rafinejad et al. 2023). Although it is a standard treatment in humans, some studies demonstrated that this dosage in suspectable BALB/c can limit lesion progression without complete healing when injected IM or IP. As shown in Figs 1- 4, Glucantime therapy at 20 mg sb^5^/kg limits the lesion size without complete healing; however, a significant reduction in lesion size was observed in groups that received chemotherapy compared to untreated control.

**Fig. 1.** The BALB/c mice were injected subcutaneously at the left footpad using 2 × 10^6^ stationary-phase of *Leishmania major* promastigotes. The lesions were treated topically by niosomal combination gel twice a day for 4 weeks. Glucantime was intramuscularly (IM) administered at a dose of 20 Sb^5^mg/kg/day. After 8 weeks, the lesion size was reduced without complete healing (Mostafavi, Sharifi et al. 2019).

**Fig 2:** Progress of lesion size in inoculated BALB/c mice by L. major in study groups at the 6^th^ week. In Control/Glucantime (G8), after 6 weeks of treatment, the lesion size was between 2.5-12.5 mm^2^ (Rahimi, Rafinejad et al. 2023).

**Fig. 3**: Lesion size in *L. major* infected BALB/c mice treated with glucantime (20 sb^5^mg/kg) alone or with silymarin (25 mg/kg) and the untreated control group. (Mean ± SEM, n = 6). The tendency of lesion size is progressive; however, chemotherapy plus supplements can reduce the lesion size compared to untreated control (Jabini, Jaafari et al. 2015).

**Fig. 4.** Progress of lesion size in *L. major* infected BALB/c mice treated with different formulations of topical liposomal MA. The lesions were treated topically with 50 mg formulations twice a day for 4 weeks. Although the lesion size was gradually increased, they were significantly smaller than that of untreated groups after termination of the treatment. (Lip-MA: liposomal meglumine antimoniate; SA: Stearylamine) (Moosavian, Fallah et al. 2019).

This trend in response to Glucantime therapy in BALB/c was also detected in other *Leishmania* species (Godinho, Simas-Rodrigues et al. 2012, Gonçalves-Oliveira, Souza-Silva et al. 2019). As shown in Figs 5 and 6, meglumine antimoniate at a dosage of 50 mg/kg limits the lesion progression; however, it causes a reduction in lesion size compared to untreated control.

**Fig. 5.** *In vivo* efficacy of Glucantime^®^ (50 mg/kg/day) and different dosages of oral miltefosine (MLT) treatment in BALB/c mice infected with *L. amazonensis* (Godinho, Simas-Rodrigues et al. 2012).

**Fig. 6**. Growth of the lesion size in BALB/c mice infected with *L. amazonensis* for 12 weeks. (a) Intralesional treatment with MI compounds or meglumine antimoniate (28 mg of Sb ^5+^ /Kg/day) from 4 to 8 weeks post-infection (Rodrigues, Charret et al. 2012).

In some studies, a higher dose of antimonial (100 mg Sb^5^/kg) with a longer period of treatment, up to 60 days, was used to achieve partial complete wound healing regardless of possible toxicity (Tiuman, Brenzan et al. 2012, Ramos, Silva et al. 2020, Sombra Santos, de Castro Rodrigues et al. 2023). As the higher dosage of systemic injection of Glucantime (intramuscular or peritoneal injection) has toxicity such as nephrotoxic side effects, we choose the standard dosage. Moreover, to ascertain the possible effect of EGF, we also used topical nano liposomal Amphotericin B beside the Glucantime. We ended the treatment after 5 weeks because we achieved a significant difference between the groups that received EGF 4.5 µg/kg compared to other groups; also, the lesion size in untreated control reached about 20 mm^2,^ and some of the mice were dead in the next week so we ended up the treatment schedule at the end of the fifth week.

We suggested that a higher dose of Glucantime and an extension of treatment duration can be used in future studies and considered as the limitation of this study (highlighted in green).

**2. Generally, 50-75% of CL lesions due to L. major heal spontaneously within 4-6 months. How to ascertain that self-healing didn't take place in any of the groups.**

**Response:** Thanks, the reviewer, for the comments; we agree that many cases of human CL due to *L. major* are spontaneously healed within 4-6 months.

However, BALB/c strains that mount a Th2 response after infection display an acute nonhealing phenotype because of an inability to produce requisite high levels of IFN-γ. Indeed, the susceptibility of BALB/c mice has been correlated to the predominance of Th2 immune response, characterized by the production of high levels of IL-4, IL-10, and IL-17 in response to infections with this parasite. Therefore, BALB/c is extremely susceptible to *L. major* that induces progressive nonhealing lesions and uniformly fatal disseminating visceral disease even with minimal infecting doses (Heinzel, Sadick et al. 1989, Müller, Pedrazzini et al. 1989, Sacks and Noben-Trauth 2002, Von Stebut, Ehrchen et al. 2003, Scott, Artis et al. 2004). It should be noted that BALB/c is the animal model typically used for drug evaluation and determining the effectiveness of the drug in inhibiting lesion progression (Loeuillet, Bañuls et al. 2016, Novais, Amorim et al. 2021).

In contrast, C57BL/6 and C3H mice are resistant to *L. major* infections with a predominance of Th1 type response and typically the production of high levels of IFN-γ but no IL-4 and IL-10 (Heinzel, Sadick et al. 1989, Müller, Pedrazzini et al. 1989, Sacks and Noben-Trauth 2002, Von Stebut, Ehrchen et al. 2003, Scott, Artis et al. 2004). In C57BL/6 and C3H mice, *L. major* develop limited self-healing or non-progressive lesions (Liew and Howard 1985, Sacks and Noben-Trauth 2002, Von Stebut, Ehrchen et al. 2003, Kurey, Kobets et al. 2009).

Accordingly, numerous studies have used BALB/c as a susceptible model for *in-vivo* infectivity and drug sensitivity assessment of cutaneous leishmaniasis (Fortin, Caridha et al. 2014, Masic, Valencia Hernandez et al. 2015, Guimarães, Gomes et al. 2021, Badirzadeh, Najm et al. 2022). The following graphs represent the CL progression due to *L. major* in the BALB/c mice.

**Fig 7.** Time course of experimental cutaneous leishmaniasis in susceptible (BALB/c) (□), resistance (C57BL/6) (■), and IL-4-/- (Δ) mice. Differences in lesion size between BALB/c and C57BL/6 mice were significant (p < 0.001) from week 8 onward, whereas differences in lesion sizes between BALB/c and IL-4-/- (p < 0.001) and between C57BL/6 and IL-4-/- (p < 0.05) were significant from week 5 onward (A). Macroscopic aspect of the lesions (12 weeks post-infection) in BALB/c (B), C57BL/6 (C), and IL-4-/- (D) mice (Cangussú, Souza et al. 2009).

**Fig 8**. The progression of lesion size in CL in two suspectable (BLAB/c), IL-17-deficient BALB/c mice (IL-17^-/-^) and resistant (C57BL/6) animal models. In BALB/c mice, the lesion size gradually increased after 2 weeks of post-infection, while in C57BL/6 mice, after a slight increase within 2 weeks, the size reduced after 6-week and complete healing was achieved after 8 weeks. Kostla et al. investigated the role of IL-17 in the progression of CL in BALB/c. They demonstrated that the lesion sizes in infected IL-17-deficient BALB/c mice (IL-17^-/-^) were dramatically smaller and failed to progress compared to those in BALB/c mice. However, complete healing was not achieved even after 10 weeks (Lopez Kostka, Dinges et al. 2009).

Likewise, in the current study, lesion sizes and parasite load were remarkably increased with no self-healing in the untreated and Normal Saline control groups. On the other hand, the groups receiving anti-leishmanial drugs exhibited a more significant reduction in lesion size. We added the susceptibility of BALB/c to *L. major* with a reference in the M&M, highlighted in gray.

**3. Although there is a reduction in the lesion size in the two intervention groups (S+E4.5 and G+E4.5) but complete healing of the lesions is not seen in any of the lesions. Authors need to provide clarification for that.**

**Response:** As mentioned in the first question, we used the BALB/c mice, which are susceptible to *L. major* and develop nonhealing lesions (Sacks and Noben-Trauth 2002, Von Stebut, Ehrchen et al. 2003, Kurey, Kobets et al. 2009). According to the previous reports, treatment with 20 mg sb^5^/kg Glucantime (Figs 1-6) or topical nano liposomal amphotericin B can limit lesion progression and reduce the lesion size (Jaafari, Hatamipour et al. 2019, Alves, Arcanjo et al. 2020) (Figs 9).

Fig 9: Clinical evaluation of BALB/c mice infected with *L. major* and treated with topical formulations including Amphotericin B (Amph B). After 40 days of infection, the animals were treated twice a day for 21 days. A reduction in lesions was observed in animals treated with topical formulations containing Amph B, GA, Amph B + GA, EA, and Amph B + EA compared to untreated control. (GA: Gallic acid; EA: Ellagic acid) (Alves, Arcanjo et al. 2020). Topical Amphotericin B limits the lesion progression and reduces the lesion size compared to untreated control without complete healing.

In the present study, although the reduction in lesion size in the treated groups was apparent compared to untreated groups, the lesion was still present after a 5-week treatment, which is in harmony with previous reports (Jabini, Jaafari et al. 2015, Jaafari, Hatamipour et al. 2019, Moosavian, Fallah et al. 2019, Mostafavi, Sharifi et al. 2019, Alves, Arcanjo et al. 2020, Rahimi, Rafinejad et al. 2023). However, as shown in Fig 1C in the manuscript, the lesion in the groups that received antileishmanial plus EGF 4.5 µg/kg was smaller, which was statistically significant compared to monotherapy (*p*<0.01). Also, epithelialization was detected at the edge of the lesion compared to monotherapy with Glucantime or amphotericin B. Of note, in the group treated with SinaAmfoleish plus EGF 4.5 µg/kg, in the pathological section, there was almost no trace of the presence of the wound, inflammatory cells, and amastigote bodies representing the starting of wound-repairing. These findings support that the lesion was in the process of wound healing.

Moreover, as stated previously, we used Glucantime at a dosage of 20 mg Sb^5^/kg for 21 days based on the standard protocol for treating human CL due to *L. major*. In this line, several researchers used this standard dosage as a standard in BALB/c (Figs 1-9) (Jabini, Jaafari et al. 2015, Jaafari, Hatamipour et al. 2019, Moosavian, Fallah et al. 2019, Mostafavi, Sharifi et al. 2019, Alves, Arcanjo et al. 2020, Rahimi, Rafinejad et al. 2023). Also, as the higher dosage of systemic injection of Glucantime, the intramuscular or peritoneal injection has toxicity such as nephrotoxic side effects, so we selected this standard dosage. However, recently, it was demonstrated that Glucantime at higher doses of 100 mg/kg for a longer course in animal models can achieve complete healing regardless of possible toxicity (Brustolin, Franzói et al. 2022).

We mentioned using Glucantime at a dosage of 20 mg Sb^5+^/kg for 21 days as a limitation of this work and suggested that a higher dosage with a more extended treatment period is probably needed for complete wound healing (highlighted in green).

**4. As per the Manual for case management of cutaneous leishmaniasis in the WHO Eastern Mediterranean Region, lesions due to L.major can be treated with Cryo-or-Thermo therapy plus intralesional antimonials or topical paromomycin. However, authors have not mentioned or provide clarification of not using these interventions in any of the groups.**

**Response:** We appreciate the reviewer for raising this point. As the reviewer mentioned, in the last decade, many studies have applied cryotherapy or thermo therapy alone or plus meglumine antimoniate or other anti-*Leishmania* drugs such as paromomycin on human CL with favorable results.

We should mention that in Iran, Cryo-or-Thermo therapy alone or plus meglumine antimoniate is used for anthroponotic CL (ACL) due to *L. tropica*. Because ACL does not usually have a desirable response to Glucantime, physical treatments are more common as a complementary beside chemotherapy in this form (Hadighi, Mohebali et al. 2006, Hadighi, Boucher et al. 2007, Aflatoonian, Sharifi et al. 2019). On the other hand, since CL caused by *L. major* usually induces open, infected, and multiple skin lesions with a favorable response to intra-lesional injection of Glucantime and other anti-*Leishmania* drugs such as Ampholeish, Paromomycin, Physical treatments are less used for this form of CL in Iran. Regarding paromomycin, although it is used as the alternative therapy, in some clinical trials in Iran, the success rate of treatment with paromomycin was less than that with intralesional Glucantime (Faghihi and Tavakoli-kia 2003, Moosavi, Nakhli et al. 2005, Heras-Mosteiro, Monge-Maillo et al. 2017).

Accordingly, Glucantime is still the first choice for treating CL due to *L. major* and *L. tropica* in Iran. In addition, recently, a new formulation of topical nano liposomal amphotericin B has been recommended for the treatment with Glucantime, and in phase 2 clinical trial, it showed a promising efficacy of 98% (Jaafari, Hatamipour et al. 2019, Khamesipour, Mohammadi et al. 2022, Alizadeh, Shirzadi et al. 2023). In the present study, we aimed to discover the effect of EGF as a complementary treatment to these available drugs in accelerating the treatment of CL.

It should be stated that because EGF was injected subcutaneously on alternate days, we could not use intralesional injection of Glucantime or Cryo-or-Thermo therapy to avoid the extra manipulation of the lesion and probable side effects such as necrosis, localized pruritus, which could affect the results (Esfandiarpour, Farajzadeh et al. 2012, Brito, Machado de Assis et al. 2019). Also, in several studies, intramuscular injection of Glucantime was used in CL animal models (Henao, Osorio et al. 2004, Tiuman, Brenzan et al. 2012, Mohtasebi, Mohebali et al. 2019).

Since the main objective of this study was the evaluation of EGF as a supplement in the treatment of cutaneous leishmaniasis caused by *Leishmania major* in BALB/c mice, we used two treatments used for CL caused by *L.major* in Iran to evaluate the complementary effect of EGF.

We suggested that a combination of EGF with other treatment regimens, such as cryo-or-thermo therapy or topical paromomycin, can also be evaluated in future studies. We added this statement as a suggestion in the discussion (highlighted in yellow).

Furthermore, in the introduction section, we add cryo-or-thermo therapy plus intralesional antimonials or topical paromomycin as an alternative treatment for CL (written in blue).

**5. Introduction section- in the first paragraph, there is a need to revise the global situation of CL and number of endemic countries. Authors can refer to WHO's recent publication of Global Leishmaniasis Surveillance Updates 2022 published in WHO weekly epidemiological record.**

**Respond:** The introduction was revised with the relevant references (written in red).

**References**

Aflatoonian, M. R., I. Sharifi, B. Aflatoonian, M. Bamorovat, A. Heshmatkhah, Z. Babaei, P. Ghasemi Nejad Almani, M. A. Mohammadi, E. Salarkia, A. Aghaei Afshar, H. Sharifi, F. Sharifi, A. Khosravi, M. Khatami, N. Arefinia, A. Fekri, S. Farajzadeh, A. Khamesipour, M. Mohebali, M. M. Gouya, M. R. Shirzadi and R. S. Varma (2019). "Associated-risk determinants for anthroponotic cutaneous leishmaniasis treated with meglumine antimoniate: A cohort study in Iran." PLoS Negl Trop Dis **13**(6): e0007423.

Alizadeh, Z., M. R. Shirzadi, G. R. Hassanpour, H. Keshavarz, F. Mohebali, S. E. Eskandari, M. Zeinali, S. Shirmohammad and M. Mohebali (2023). "Field Efficacy of Topical Nano-Liposomal Amphotericin B (Sina Ampholeish®) Alone or in Combination with Glucantime® and Cryotherapy on Human Cutaneous Leishmaniasis." Iran J Parasitol **18**(4): 419-426.

Alves, M. M. M., D. D. R. Arcanjo, K. A. Figueiredo, J. Oliveira, F. J. C. Viana, E. S. Coelho, G. L. N. Lopes, J. C. R. Gonçalves, A. L. M. Carvalho, M. D. S. Rizzo, M. H. Chaves, I. L. Mendonça and F. A. A. Carvalho (2020). "Gallic and Ellagic Acids Are Promising Adjuvants to Conventional Amphotericin B for the Treatment of Cutaneous Leishmaniasis." Antimicrob Agents Chemother **64**(12).

Badirzadeh, A., M. Najm, A. Hemphill, M. Alipour, H. Hasanpour, L. Masoori and P. Karimi (2022). "Effects of negative air ions (NAIs) on Leishmania major: A novel tool for treatment of zoonotic cutaneous leishmaniasis (ZCL)." PLoS One **17**(9): e0274124.

Brito, N. C., T. S. Machado de Assis, A. Rabello and G. Cota (2019). "Intralesional infiltration versus parenteral use of meglumine antimoniate for treatment of cutaneous leishmaniasis: A cost-effectiveness analysis." PLoS Negl Trop Dis **13**(12): e0007856.

Brustolin, A., N. M. Franzói, C. F. H. Ramos-Milaré Á, C. A. Tanoshi, C. A. Mota, I. G. Demarchi, M. V. C. Lonardoni and T. G. Verzignassi Silveira (2022). "A standardized intraperitoneal Glucantime™ for experimental treatment of cutaneous leishmaniasis caused by Leishmania amazonensis in BALB/c mice." Exp Parasitol **236-237**: 108259.

Cangussú, S. D., C. C. Souza, C. F. Campos, L. Q. Vieira, L. C. Afonso and R. M. Arantes (2009). "Histopathology of Leishmania major infection: revisiting L. major histopathology in the ear dermis infection model." Mem Inst Oswaldo Cruz **104**(6): 918-922.

Esfandiarpour, I., S. Farajzadeh, Z. Rahnama, E. A. Fathabadi and A. Heshmatkhah (2012). "Adverse effects of intralesional meglumine antimoniate and its influence on clinical laboratory parameters in the treatment of cutaneous leishmaniasis." Int J Dermatol **51**(10): 1221-1225.

Faghihi, G. and R. Tavakoli-kia (2003). "Treatment of cutaneous leishmaniasis with either topical paromomycin or intralesional meglumine antimoniate." Clin Exp Dermatol **28**(1): 13-16.

Fortin, A., D. P. Caridha, S. Leed, F. Ngundam, J. Sena, T. Bosschaerts, S. Parriott, M. R. Hickman, T. H. Hudson and M. Grogl (2014). "Direct comparison of the efficacy and safety of oral treatments with oleylphosphocholine (OlPC) and miltefosine in a mouse model of L. major cutaneous leishmaniasis." PLoS Negl Trop Dis **8**(9): e3144.

Godinho, J. L., C. Simas-Rodrigues, R. Silva, T. P. Ürmenyi, W. de Souza and J. C. Rodrigues (2012). "Efficacy of miltefosine treatment in Leishmania amazonensis-infected BALB/c mice." Int J Antimicrob Agents **39**(4): 326-331.

Gonçalves-Oliveira, L. F., F. Souza-Silva, L. M. de Castro Côrtes, L. B. Veloso, B. A. Santini Pereira, L. Cysne-Finkelstein, G. C. Lechuga, S. C. Bourguignon, F. Almeida-Souza, K. da Silva Calabrese, V. F. Ferreira and C. R. Alves (2019). "The combination therapy of meglumine antimoniate and oxiranes (epoxy-α-lapachone and epoxymethyl-lawsone) enhance the leishmanicidal effect in mice infected by Leishmania (Leishmania) amazonensis." Int J Parasitol Drugs Drug Resist **10**: 101-108.

Guimarães, T. T., S. M. R. Gomes, R. Albuquerque, A. K. C. Lima, G. F. Braga, J. B. Souza, M. Assis, A. C. S. Brito, R. F. Santos, T. Da Silva, L. M. Siqueira, B. D. Ventura, L. S. Rodrigues, R. Terra, S. A. G. Da Silva and P. M. L. Dutra (2021). "Chronic Aerobic Training at Different Volumes in the Modulation of Macrophage Function and in vivo Infection of BALB/c Mice by Leishmania major." Front Microbiol **12**: 734355.

Hadighi, R., P. Boucher, A. Khamesipour, A. R. Meamar, G. Roy, M. Ouellette and M. Mohebali (2007). "Glucantime-resistant Leishmania tropica isolated from Iranian patients with cutaneous leishmaniasis are sensitive to alternative antileishmania drugs." Parasitol Res **101**(5): 1319-1322.

Hadighi, R., M. Mohebali, P. Boucher, H. Hajjaran, A. Khamesipour and M. Ouellette (2006). "Unresponsiveness to Glucantime treatment in Iranian cutaneous leishmaniasis due to drug-resistant Leishmania tropica parasites." PLoS Med **3**(5): e162.

Heinzel, F. P., M. D. Sadick, B. J. Holaday, R. L. Coffman and R. M. Locksley (1989). "Reciprocal expression of interferon gamma or interleukin 4 during the resolution or progression of murine leishmaniasis. Evidence for expansion of distinct helper T cell subsets." J Exp Med **169**(1): 59-72.

Henao, H. H., Y. Osorio, N. G. Saravia, A. Gómez and B. Travi (2004). "[Efficacy and toxicity of pentavalent antimonials (Glucantime and Pentostam) in an American cutaneous leishmaniasis animal model: luminometry application]." Biomedica **24**(4): 393-402.

Heras-Mosteiro, J., B. Monge-Maillo, M. Pinart, P. Lopez Pereira, L. Reveiz, E. Garcia-Carrasco, P. Campuzano Cuadrado, A. Royuela, I. Mendez Roman and R. López-Vélez (2017). "Interventions for Old World cutaneous leishmaniasis." Cochrane Database Syst Rev **12**(12): Cd005067.

Jaafari, M. R., M. Hatamipour, S. H. Alavizadeh, A. Abbasi, Z. Saberi, S. Rafati, Y. Taslimi, A. M. Mohammadi and A. Khamesipour (2019). "Development of a topical liposomal formulation of Amphotericin B for the treatment of cutaneous leishmaniasis." Int J Parasitol Drugs Drug Resist **11**: 156-165.

Jabini, R., M. R. Jaafari, F. Vahdati Hasani, F. Ghazizadeh, A. Khamesipour and G. Karimi (2015). "Effects of combined therapy with silymarin and glucantime on leishmaniasis induced by Leishmania major in BALB/c mice." Drug Res (Stuttg) **65**(3): 119-124.

Khamesipour, A., A. Mohammadi, M. Jaafari, S. Eskandari, M. Tasbihi, A. Javadi, F. Afshari, H. Mortazavi and A. Firooz (2022). "Pilot study of safety and efficacy of topical liposomal amphotericin B for cutaneous leishmaniasis caused by Leishmania major in Islamic Republic of Iran." East Mediterr Health J **28**(9): 658-663.

Kurey, I., T. Kobets, H. Havelková, M. Slapnicková, L. Quan, K. Trtková, I. Grekov, M. Svobodová, A. P. Stassen, A. Hutson, P. Demant and M. Lipoldová (2009). "Distinct genetic control of parasite elimination, dissemination, and disease after Leishmania major infection." Immunogenetics **61**(9): 619-633.

Liew, F. Y. and J. G. Howard (1985). "Role of T cells in the unusual cutaneous responses to Leishmania in BALB/c mice." Curr Top Microbiol Immunol **122**: 122-127.

Loeuillet, C., A. L. Bañuls and M. Hide (2016). "Study of Leishmania pathogenesis in mice: experimental considerations." Parasit Vectors **9**: 144.

Lopez Kostka, S., S. Dinges, K. Griewank, Y. Iwakura, M. C. Udey and E. von Stebut (2009). "IL-17 promotes progression of cutaneous leishmaniasis in susceptible mice." J Immunol **182**(5): 3039-3046.

Masic, A., A. M. Valencia Hernandez, S. Hazra, J. Glaser, U. Holzgrabe, B. Hazra and U. Schurigt (2015). "Cinnamic Acid Bornyl Ester Derivatives from Valeriana wallichii Exhibit Antileishmanial In Vivo Activity in Leishmania major-Infected BALB/c Mice." PLoS One **10**(11): e0142386.

Mohtasebi, S., M. Mohebali, S. Elikaee, B. Akhoundi, A. R. Foroushani, A. Teimouri and H. Yarizadeh (2019). "In vitro and in vivo anti-parasitic activity of biogenic antimony sulfide nanoparticles on Leishmania major (MRHO/IR/75/ER)." Parasitol Res **118**(9): 2669-2678.

Moosavi, Z., A. Nakhli and S. Rassaii (2005). "Comparing the efficiency of topical paromomycin with intralesional meglumine antimoniate for cutaneous leishmaniasis." Int J Dermatol **44**(12): 1064-1065.

Moosavian, S. A., M. Fallah and M. R. Jaafari (2019). "The activity of encapsulated meglumine antimoniate in stearylamine-bearing liposomes against cutaneous leishmaniasis in BALB/c mice." Exp Parasitol **200**: 30-35.

Mostafavi, M., I. Sharifi, S. Farajzadeh, P. Khazaeli, H. Sharifi, E. Pourseyedi, S. Kakooei, M. Bamorovat, A. Keyhani, M. H. Parizi, A. Khosravi and A. Khamesipour (2019). "Niosomal formulation of amphotericin B alone and in combination with glucantime: In vitro and in vivo leishmanicidal effects." Biomed Pharmacother **116**: 108942.

Müller, I., T. Pedrazzini, J. P. Farrell and J. Louis (1989). "T-cell responses and immunity to experimental infection with leishmania major." Annu Rev Immunol **7**: 561-578.

Nishikomori, R., S. Gurunathan, K. Nishikomori and W. Strober (2001). "BALB/c mice bearing a transgenic IL-12 receptor beta 2 gene exhibit a nonhealing phenotype to Leishmania major infection despite intact IL-12 signaling." J Immunol **166**(11): 6776-6783.

Novais, F. O., C. F. Amorim and P. Scott (2021). "Host-Directed Therapies for Cutaneous Leishmaniasis." Front Immunol **12**: 660183.

Rahimi, S., J. Rafinejad, A. A. Akhavan, R. Ahmadkhaniha, M. Bakhtiyari, A. Khamesipour and K. Akbarzadeh (2023). "The therapeutic effect of larval saliva and hemolymph of Lucilia sericata on the treatment of Leishmania major lesion in BALB/c mice946." Parasit Vectors **16**(1): 72.

Ramos, T. D., J. D. Silva, A. M. da Fonseca-Martins, J. E. da Silveira Pratti, L. Firmino-Cruz, D. Maciel-Oliveira, J. S. Dos-Santos, J. I. N. Tenorio, A. F. de Araujo, C. G. Freire-de-Lima, B. L. Diaz, F. F. Cruz, P. R. M. Rocco and H. L. de Matos Guedes (2020). "Combined therapy with adipose tissue-derived mesenchymal stromal cells and meglumine antimoniate controls lesion development and parasite load in murine cutaneous leishmaniasis caused by Leishmania amazonensis." Stem Cell Res Ther **11**(1): 374.

Rodrigues, R. F., K. S. Charret, M. C. Campos, V. Amaral, A. Echevarria, C. Dos Reis, M. M. Canto-Cavalheiro and L. L. Leon (2012). "The in vivo activity of 1,3,4-thiadiazolium-2-aminide compounds in the treatment of cutaneous and visceral leishmaniasis." J Antimicrob Chemother **67**(1): 182-190.

Sacks, D. and N. Noben-Trauth (2002). "The immunology of susceptibility and resistance to Leishmania major in mice." Nat Rev Immunol **2**(11): 845-858.

Scott, P., D. Artis, J. Uzonna and C. Zaph (2004). "The development of effector and memory T cells in cutaneous leishmaniasis: the implications for vaccine development." Immunol Rev **201**: 318-338.

Sombra Santos, A., N. L. de Castro Rodrigues, F. R. Marciano Fonseca, N. Braga Fayão Oliveira, B. Oliveira Loucard, F. Fernandes Heredia, T. N. Albuquerque Gomes Nogueira, T. Monteiro Abreu, H. Silva Dos Santos, E. Holanda Teixeira, L. K. Almeida Moreira Leal, R. B. Brandim Gomes, C. Romero Teixeira and M. J. Teixeira (2023). "Leishmanicidal and healing effects of 3β,6β,16β-trihydroxy lup-20 (29)-ene isolated from Combretum leprosum on Leishmania braziliensis infection in vitro and in vivo." PLoS One **18**(11): e0287665.

Tiuman, T. S., M. A. Brenzan, T. Ueda-Nakamura, B. P. Filho, D. A. Cortez and C. V. Nakamura (2012). "Intramuscular and topical treatment of cutaneous leishmaniasis lesions in mice infected with Leishmania amazonensis using coumarin (-) mammea A/BB." Phytomedicine **19**(13): 1196-1199.

Von Stebut, E., J. M. Ehrchen, Y. Belkaid, S. L. Kostka, K. Molle, J. Knop, C. Sunderkotter and M. C. Udey (2003). "Interleukin 1alpha promotes Th1 differentiation and inhibits disease progression in Leishmania major-susceptible BALB/c mice." J Exp Med **198**(2): 191-199.
